# Supplementary material for: A computational method for cell type-specific expression quantitative trait loci mapping using bulk RNA-seq data
Source: Nat Commun. 2023 May 25;14:3030. doi: 10.1038/s41467-023-38795-w (PMC10212972; doi:10.1038/s41467-023-38795-w)
Supplement: Supplementary file 1 — Supplementary Information [file 41467_2023_38795_MOESM1_ESM.pdf]

Supplementary Materials for "A computational method for Cell type-specific Expression Quantitative Trait Loci mapping using bulk RNA-seq data"

Paul Little, Si Liu, Vasyl Zhabotynsky, Yun Li, Dan-Yu Lin, Wei Sun

Contents

1 Extended Simulations **2**

2 Deconvolution **7**

2.1 Input/output for deconvolution 7

2.2 Considered Methods 7

2.3 LM22 transformation 7

2.4 Signature Expression from scRNA-seq 10

2.5 Collapsing cell types 12

3 Multiple Testing and FDR **12**

4 Data Preprocessing **13**

5 Quality Control, Filtering, ASReC adjustment **14**

6 Real Data Analyses **17**

6.1 Covariates 17

6.2 Commonmind Consortium 18

6.3 GTEx Brain 21

6.4 BLUEPRINT 24

6.5 GTEx Whole Blood 27

6.6 Validation 30

6.7 Consistency and enrichment 35

6.8 Summary of number of eQTL findings with alternative q-value cutoff 38

6.9 Comparison of eQTL effect directions 40

Supplementary Note 1: Extended Simulations

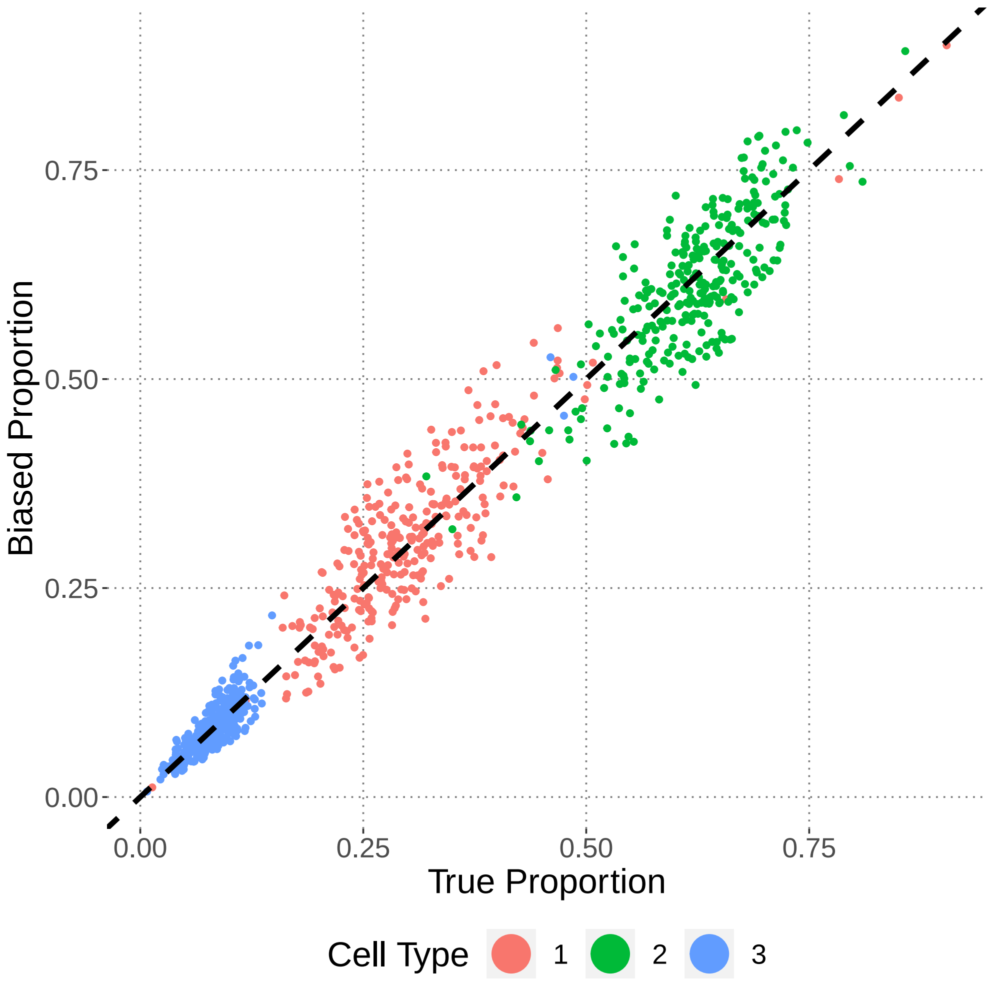

Supplementary Figure 1: Simulated cell type proportions under Scenario 3 with cell type proportions after adding noise on the y-axis and true proportions on the x-axis.

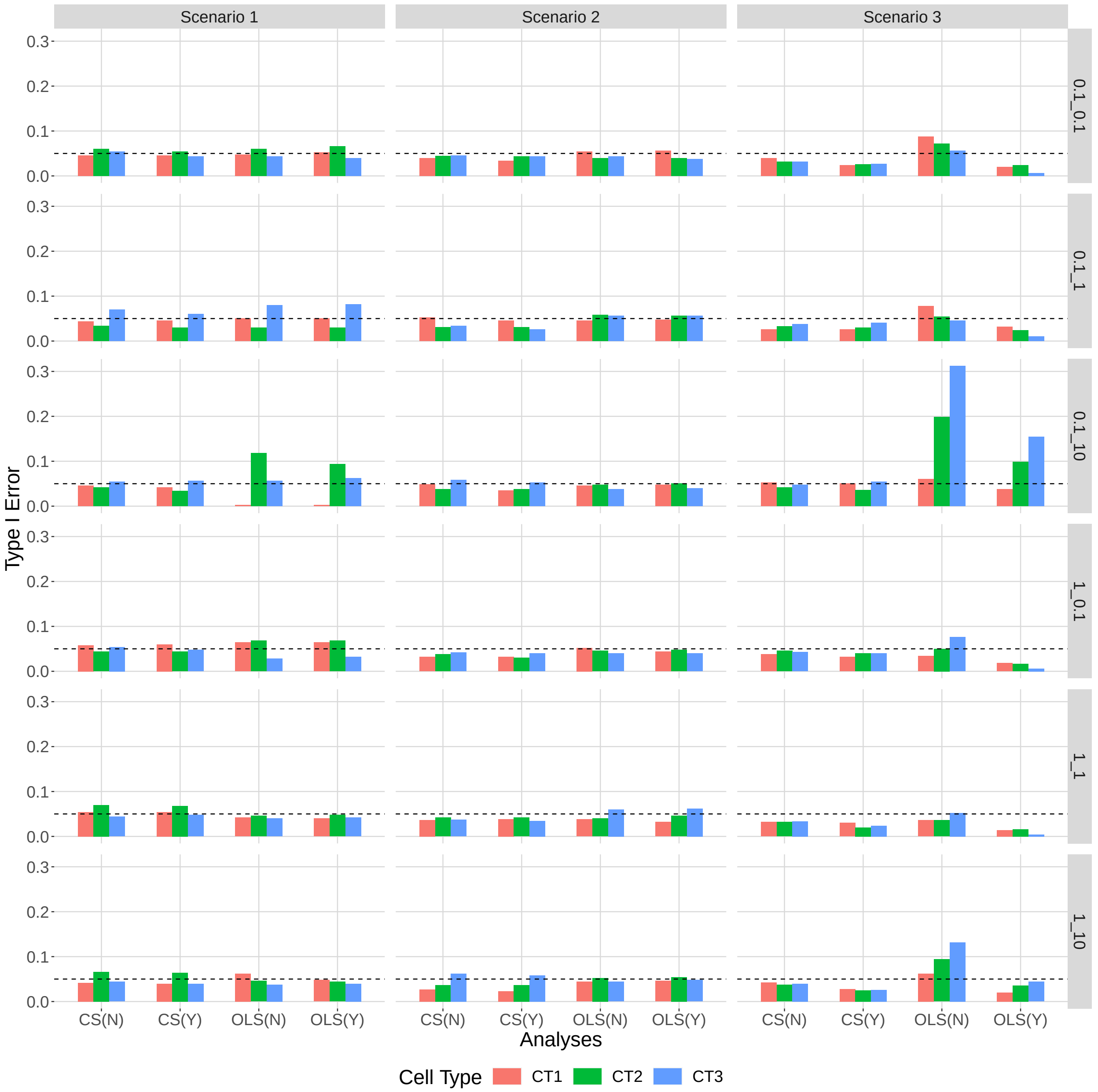

Supplementary Figure 2: Type I error for the data simulated under complete null, with **true** cell type proportions. X-axis labels CS(N), CS(Y), OLS(N), and OLS(Y) correspond to CSeQTL w/o trimming, CSeQTL w/ trimming, OLS w/o trimming, and OLS w/ trimming, respectively.

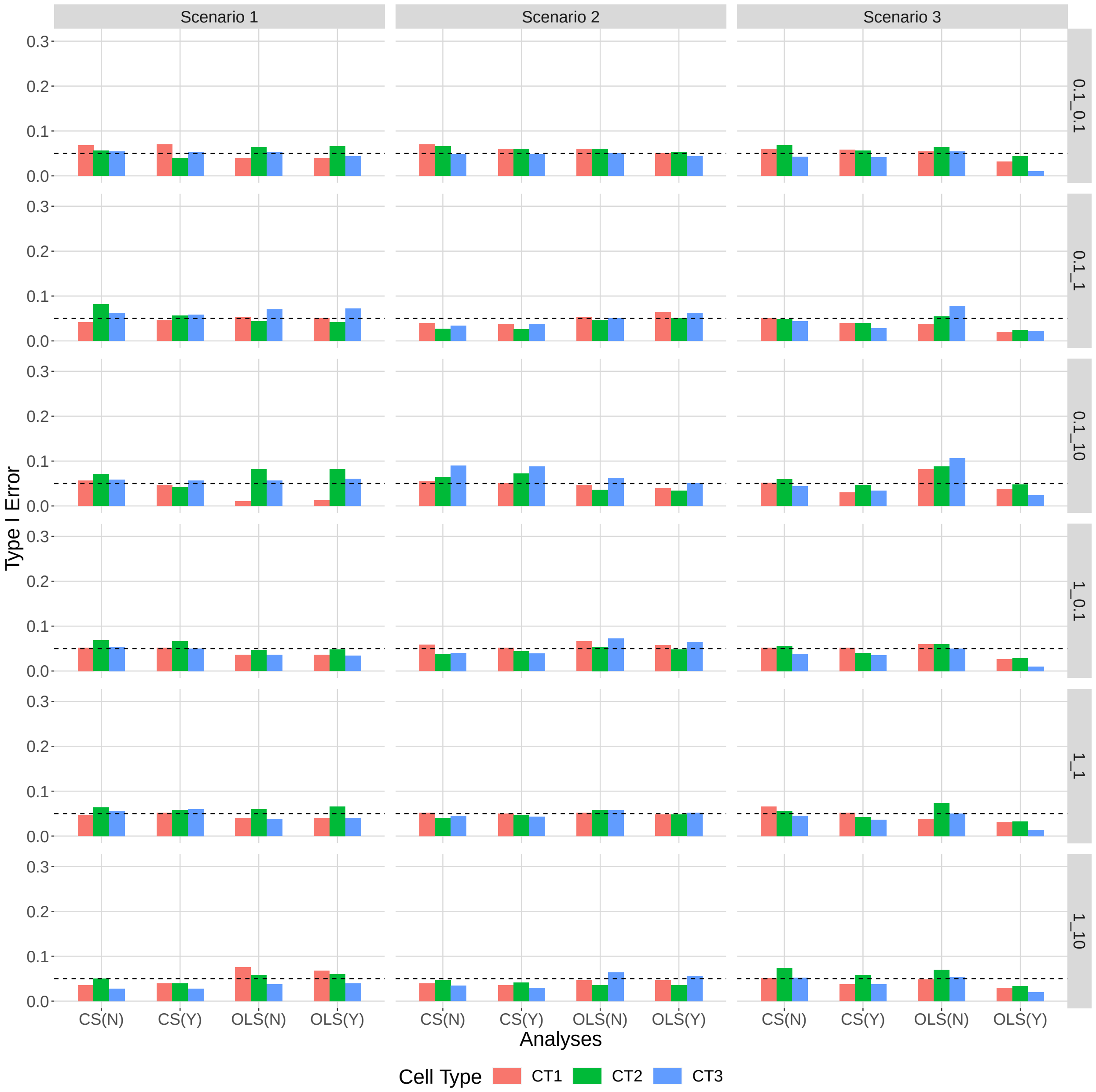

Supplementary Figure 3: Type I error for the data simulated under complete null, with **noisy** cell type proportions. X-axis labels CS(N), CS(Y), OLS(N), and OLS(Y) correspond to CSeQTL w/o trimming, CSeQTL w/ trimming, OLS w/o trimming, and OLS w/ trimming, respectively.

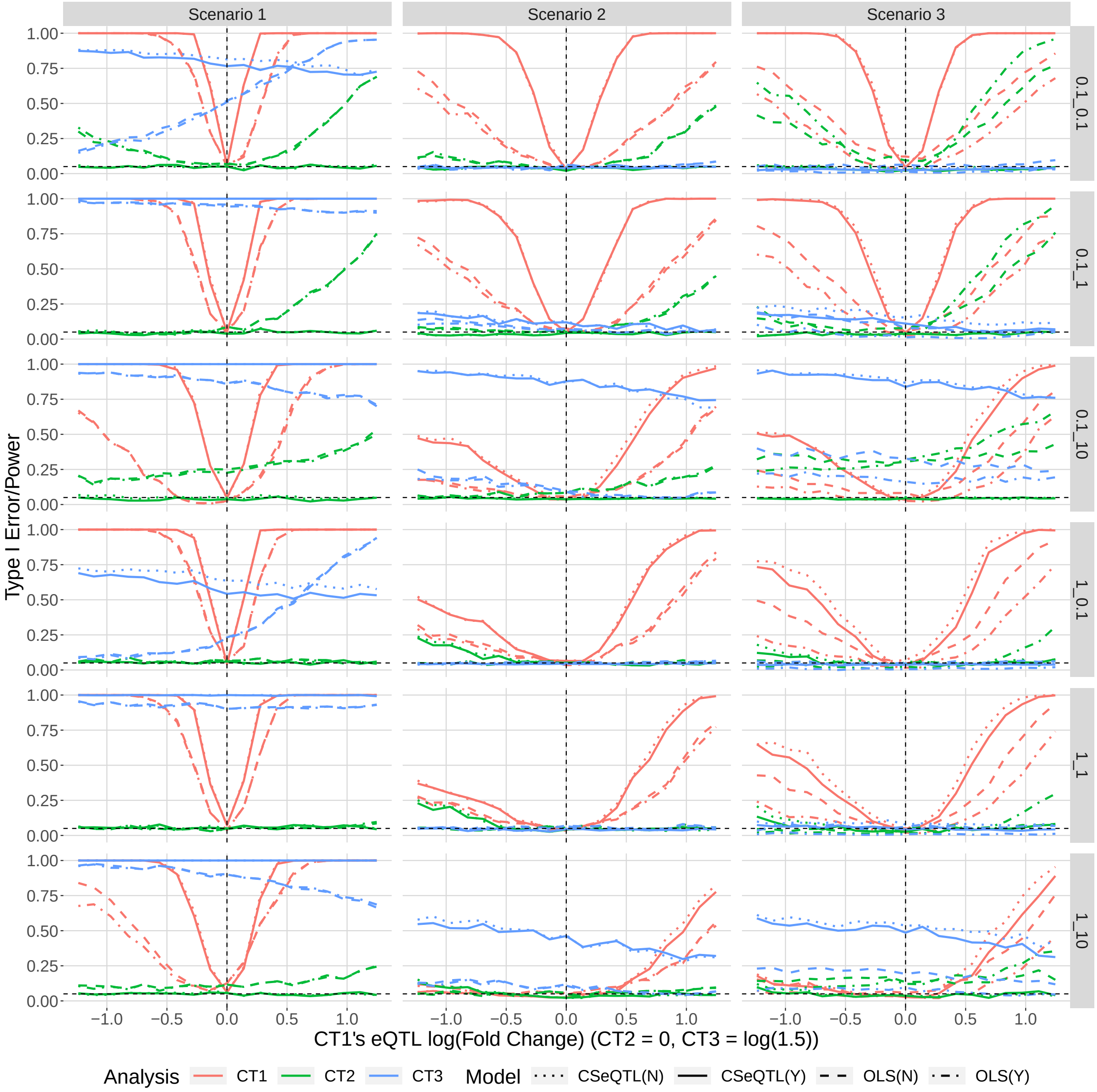

Supplementary Figure 4: Power/Type I error for the data simulated under mixed null/alternative with **true** cell type proportions. X-axis labels CSeQTL(N), CSeQTL(Y), OLS(N), and OLS(Y) correspond to CSeQTL w/o trimming, CSeQTL w/ trimming, OLS w/o trimming, and OLS w/ trimming, respectively.

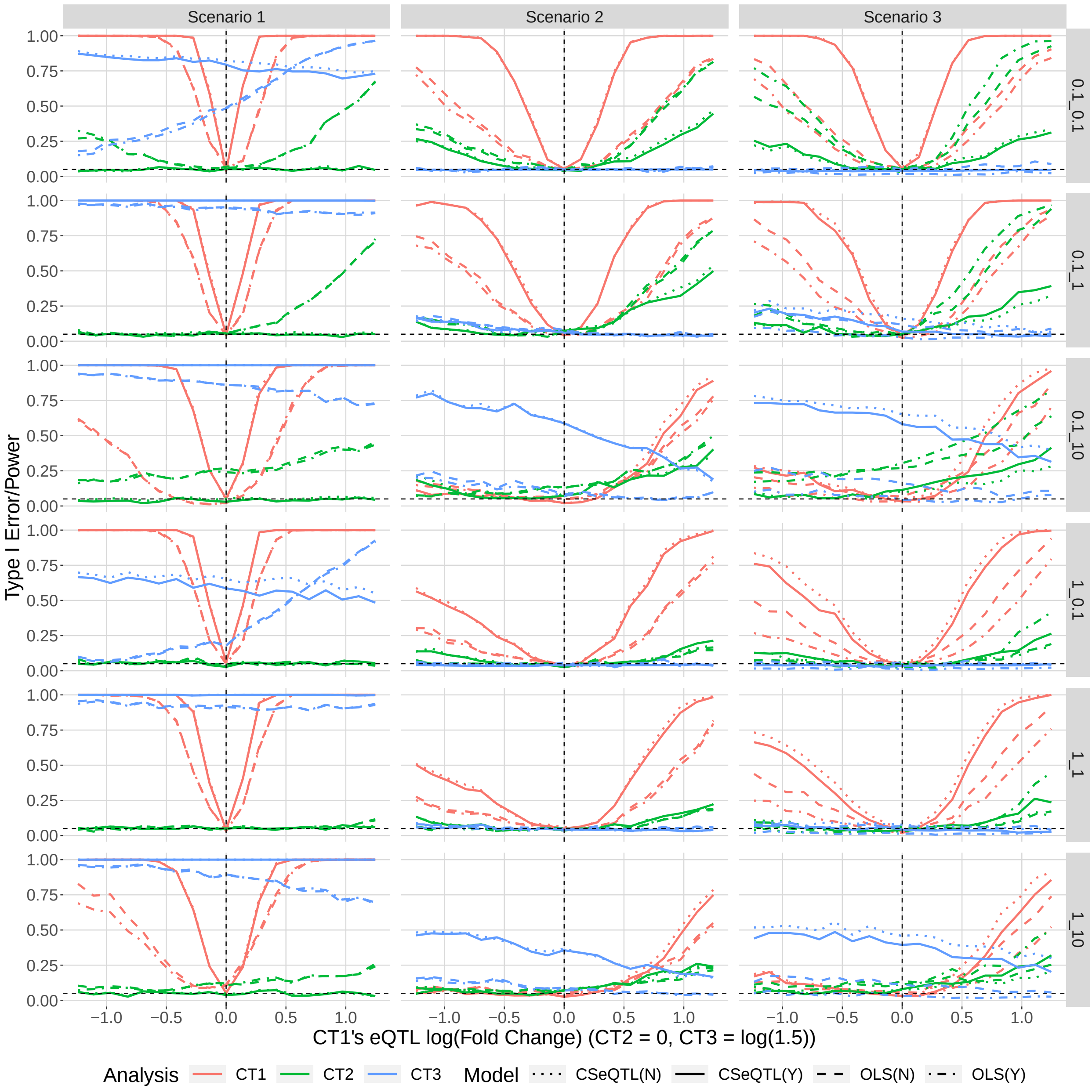

Supplementary Figure 5: Power/Type I error for the data simulated under mixed null/alternative with **noisy** cell type proportions. X-axis labels CSeQTL(N), CSeQTL(Y), OLS(N), and OLS(Y) correspond to CSeQTL w/o trimming, CSeQTL w/ trimming, OLS w/o trimming, and OLS w/ trimming, respectively.

Supplementary Note 2: Deconvolution

2.1 Input/output for deconvolution

To perform deconvolution, total read count data from the i-th bulk RNA-seq sample (T_{ig}) and reference scRNA-seq data for the qth cell type, denoted by R_{qg}, across G shared genes are converted to transcripts per million (TPM), denoted by t_{ig} and r_{qg}:

$$t_{ig} = 10^6 \frac{T_{ig}/l_g}{\sum_{h=1}^{G} T_{ih}/l_h} \text{ and } r_{qg} = 10^6 \frac{R_{qg}/l_g}{\sum_{h=1}^{G} R_{qh}/l_h},$$

where l_g denotes the gth gene's exonic region length. For reference data from scRNA-seq, we can calculate cell size factors for the qth cell type, denoted s_q and it is proportional to $\sum_{g=1}^{G}(R_{qg}/l_g)$. For ease of comparison across other studies, we calculate $s_q = \sum_{g=1}^{G}(R_{qg}/l_g)$ and then normalize it by the largest cell type's cell size ($s_q = s_q/\max_r(s_r)$). For reference data not derived from scRNA-seq, we use cell size factors reported by other studies. We estimate cell type proportions using ICeDT [1], with TPM data as input, setting tumor purity to 0 for all samples, and specifying default values for the remaining arguments. Cell type proportion estimates per sample, denoted by $\tilde{\rho}_{iq}$, are considered proportions of transcripts per cell type that are then corrected using cell size factors, such that the final cell type proportion estimates are given by

$$\rho_{iq} = \frac{\tilde{\rho}_{iq}/s_q}{\sum_{r=1}^{Q} \tilde{\rho}_{ir}/s_r}.$$

For CMC and GTEx brain cohorts, we corrected for cell size factors from MTG (astrocyte = 0.885, excitatory = 0.893, inhibitory = 1.00, microglia = 0.856, oligodendrocyte = 0.927, oligodendrocyte precursor cells = 0.726). For whole blood samples we used cell sizes reported by EPIC [2], which have been utilized in our earlier work [1].

2.2 Considered Methods

Many methods have been developed to estimate cell type compositions and most of them rely on cell type-specific expression from external sources, such as scRNA-seq or purified samples of individual cell types [3, 4]. These methods differ in their models and strategies to improve their robustness and accuracy. For example, CIBERSORT [4] employs support-vector regression and its relu loss function is robust to outliers. TIMER [5] uses a linear regression with least squared loss function, and thus is sensitive to outliers. Their strategy is to remove the genes with very high expression due to their strong influence on model fitting. EPIC [2] uses weighted linear regression to give the genes with lower expression variation higher weights. ICeDT [1], which shares some similar spirit with an earlier method DeMix [6], models the gene expression by a log-normal distribution to reduce the impact of genes with high expression and conducts deconvolution in the linear scale. Importantly, ICeDT acknowledges the fact that for some genes, cell type-specific expression from external sources may not be consistent with their expression in bulk tissue samples. ICeDT uses a mixture model to down-weigh the contribution of such genes in cell type composition estimation.

2.3 LM22 transformation

For whole blood, the existing LM22 dataset, derived from microarray data would be utilized and transformed for deconvolution with RNA-seq based expression. LM22 was obtained through the CIBERSORT website and it was a matrix of gene expression for 547 marker genes and 22 cell types. CIBERSORT performs deconvolution on centered and scaled bulk and signature gene expression, this eliminates concern on units or scaling between bulk and signature expression. However, for ICeDT deconvolution, we need to ensure that the bulk and signature expression were on the same scale. To achieve this, we transformed LM22 signatures

based on an assumption that bulk TPM from samples with very high proportions of neutrophils should strongly correlate with neutrophil expression in the LM22 matrix. We first estimate cell type proportions by CIBERSORT, with TPM-transformed bulk expression and untransformed LM22 as input. Using these estimates, we subsetted samples with estimated neutrophil proportions greater than 85%, which resulted in 80 samples. We identified a subset of neutrophil-specific marker genes who have highest expression in neutrophil and their expression in the next cell type is less than half of their expression in neutrophil. For each of the 80 samples with high neutrophil proportion, we fitted a linear model per sample (log bulk TPM vs. log neutrophil expression from LM22) using those neutrophil-specific marker genes, and retained sample-specific slopes and intercepts (Supplementary Figure 6). The average slope (1.031) and intercept (-2.32) were used to transform all the genes in LM22 matrix by multiplying the log-transformed LM22 matrix by 1.031, subtracting 2.32, and exponentiating. We assumed the transformation may leave some gene expression incompatible between bulk and signature. We then compared the updated LM22 signature with the bulk TPM for each gene. More specifically, for each gene, we calculated the proportion of samples whose bulk expression was less than the minimum cell type-specific expression, as well as the proportion of samples whose bulk expression was greater than the maximum cell type-specific expression. Using cutoffs of 15% for both quantities, we subsetted 228 genes for ICeDT deconvolution (Supplementary Figure 7).

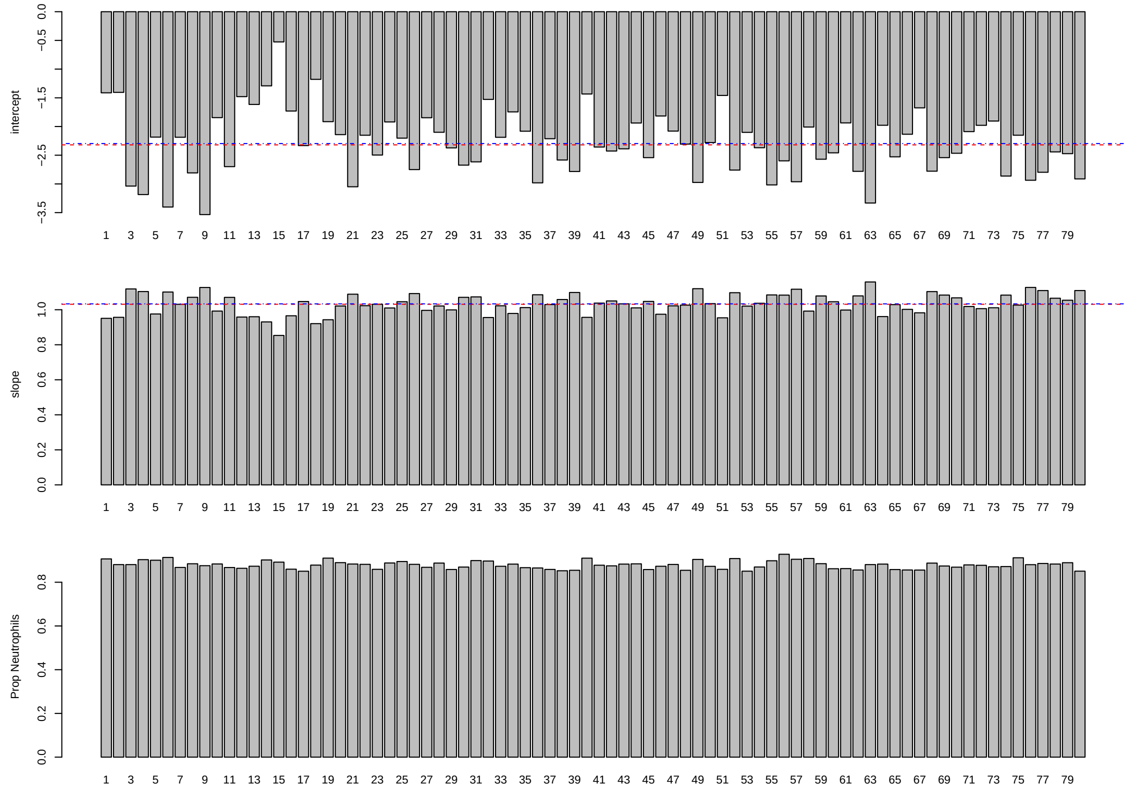

Supplementary Figure 6: **GTEx whole blood samples with greater than 85% neutrophil composition.** The first, second, and third barplots correspond to linear fit intercepts and slopes and neutrophil proportions, respectively.

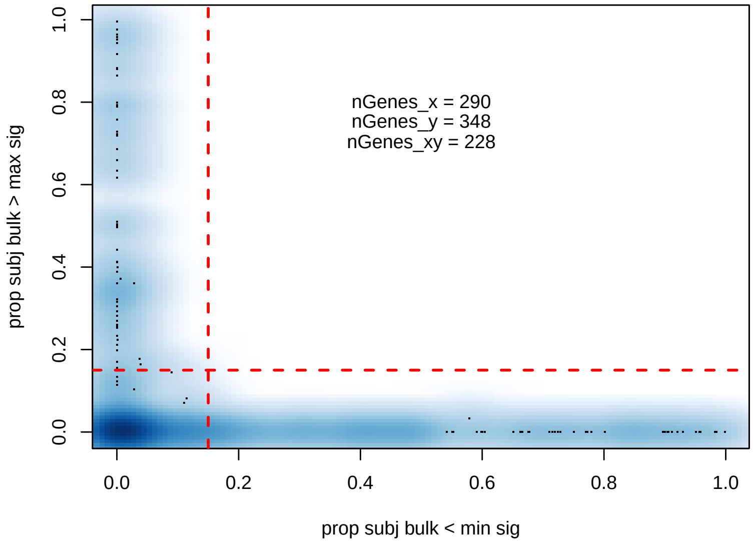

Supplementary Figure 7: **Selection of marker genes for cell types in blood.** Each point is a marker gene. The x-axis is the proportion of bulk samples where this gene's expression is smaller than the minimum expression of this gene across all cell types. The y-axis is the proportion of bulk samples where this gene's expression is larger than the maximum expression of this gene across all cell types.

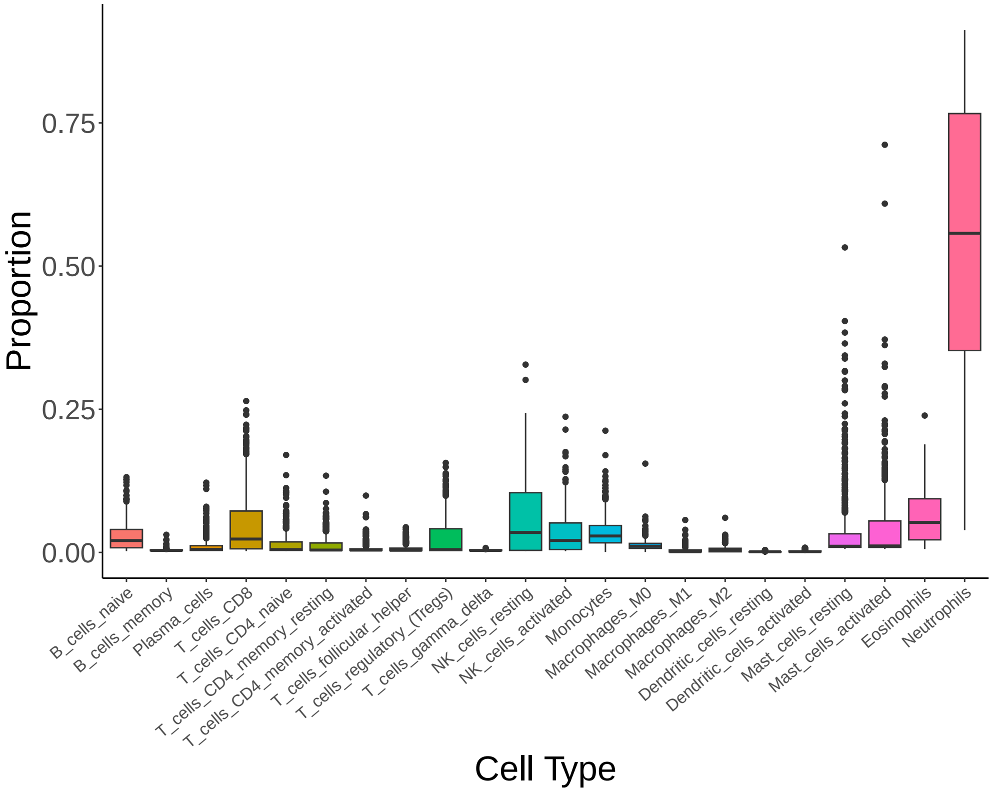

Supplementary Figure 8: **ICeDT deconvolution estimates of cell type proportion for n = 670 GTEx whole blood samples.** For each boxplot, the box ranges from Q1 (the first quartile) to Q3 (the third quartile). The median is indicated by a line across the box. The whiskers extend from Q1 and Q3 to the most extreme data points within the 1.5 IQR of the box and IQR=Q3 − Q1.

2.4 Signature Expression from scRNA-seq

To perform deconvolution on GTEx's brain frontal cortex and CMC's dorsolateral prefrontal cortex (LDPFC) samples, we studied three single nucleus RNA-seq (snRNA-seq) datasets: DroNc-seq [7] (19,550 nuclei from human brain), the Allen Brain Atlas's middle temporal gyrus (MTG) datasets [8] (15,928 nuclei by SMART-seq platform), and a very recent dataset from The Seattle Alzheimer's Disease Brain Cell Atlas (SEA-AD), which included more than 1 million nuclei from human middle temporal gyrus (MTG) of 89 donors, using 10x 3' platform. After running a workflow of quality control and clustering, we concluded not to use the DroNc-seq dataset since its clustering results were unstable, likely due to high sparsity and limited number of nuclei. In contrast, clustering results by MTG data were more robust. Different methods led to similar clusters that matched pre-labeled cell types. This was likely because MTG used the SMART-seq protocol, which generated less sparse and full-length gene expression data.

We processed MTG data using our workflow `www.github.com/Sun-lab/scRNAseq_pipelines/MTG`, which utilized R packages scater [9], scran [10], limma [11], and Rtsne [12]. When processing MTG data, 15,929 nuclei underwent quality control (QC) and filtering. We first filtered out nuclei with less than 50% unique reads, and 15,929 nuclei were retained. Next, genes were subjected to QC and filtering by retaining genes that were detected (at least 1 gene count) in at least 30 nuclei, which left us 37,657 genes in the following analysis. Normalization was conducted by calculating size factors using functions quickCluster() and computeSumFactors(). We identified a strong positive correlation between computed size factors and total counts. We used makeTechTrend() to decompose the variance of gene expression into biological and technical noise. A total of 4,745 highly variable genes were selected based on an FDR threshold of 10^{-20} and a bio threshold of 0.1, and they were used as inputs for PCA. We selected the top 50 PCs for tSNE visualization and kMeans clustering. We ran kMeans clustering with fifty initializations with specified number of clusters ranging from 10 to 20, each time comparing the clustering results to the pre-labeled cell type clusters generated by MTG. At 15 clusters, cell types and clusters had sufficient one-to-one match with each other. Among the pre-labeled cell types, only nine nuclei were labeled as endothelial and our clusters contained endothelial nuclei that overlapped strongly with OPC cell types. Thus we excluded endothelial cell type from our final reference expression calculation. We next used MAST [13] to identify approximately 120 top marker genes per cell type based on fold change and q-value. Once marker genes were identified, scRNA-seq counts were averaged within cell type to construct our signature expression matrix for subsequent bulk RNA-seq deconvolution.

We also compared gene expression similarity as well as cell type composition estimation when using MTG vs. SEA-AD data. Our results showed that the expression of signature genes were highly correlated between the two datasets and they led to very consistent cell type composition estimation (Figure 9). Therefore, the large number of nuclei available from the SEA-AD study likely overcame the sparsity limitation.

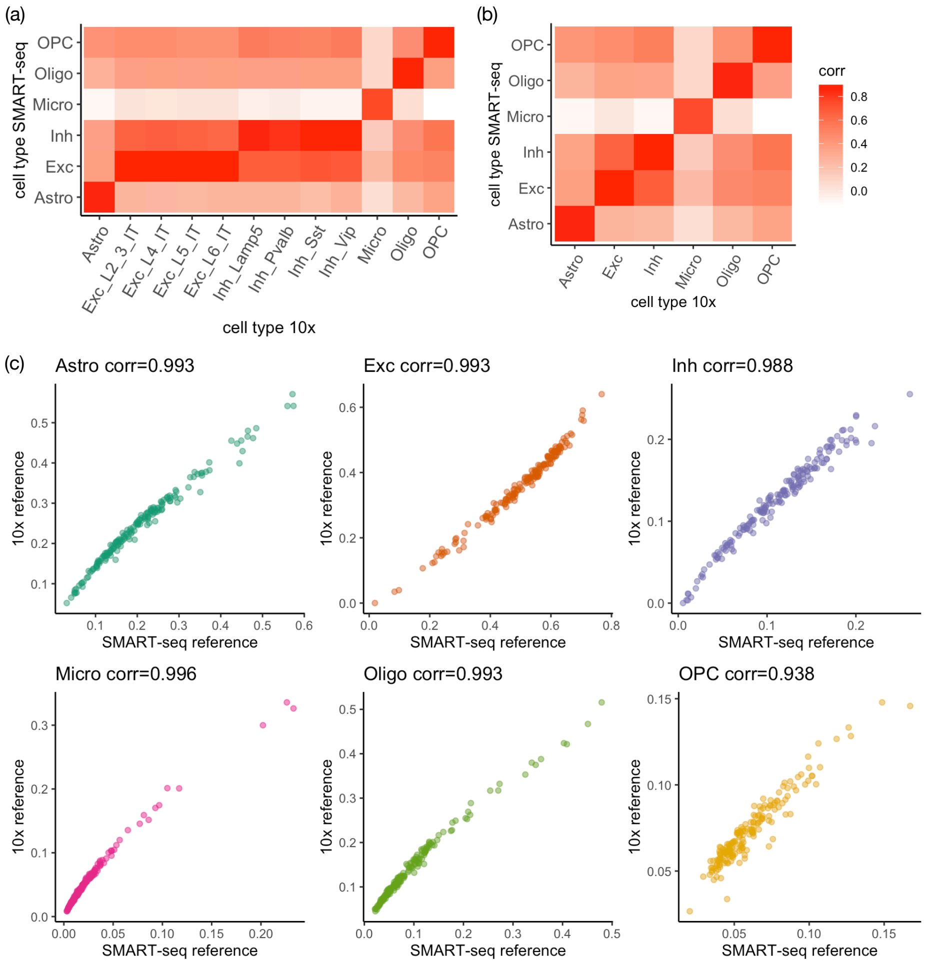

Supplementary Figure 9: **Compare cell type reference by SMART-seq vs. 10x 3' platform.** (a) Correlation of gene expression of 6 cell types from SMART-seq data versus gene expression of 12 cell types (4 sub-types of excitatory neurons labeled by Exc_L*, 4 subtypes of inhibitory neurons labeled by Inh_*, and 4 type of glia cells. (b) Similar correlation matrix as (a) except that excitatory/inhibitory neuron subtypes are combined (by adding up expression from all the cells) to excitatory/inhibitory neurons. (c). Scatterplots of cell type proportion estimates based on SMART-seq or 10x references.

2.5 Collapsing cell types

We provide an algebraic derivation of the loss of explained variability in TReC resulting from collapsing cell types. Suppose we have three cell types ($Q = 3$) and we would like to collapse cell types 1 and 2 together. We would like to interpret the parameters of the collapsed model with regard to the original parameters. Starting with the definition of the ith sample's bulk eQTL, we have

$$\xi_i = \frac{\mu_{iB}}{\mu_{iA}} = \frac{\sum_q \rho_{iq}\kappa_q\eta_q}{\sum_q \rho_{iq}\kappa_q} = \frac{\rho_{i1}\eta_1 + \rho_{i2}\kappa_2\eta_2 + \rho_{i3}\kappa_3\eta_3}{\rho_{i1} + \rho_{i2}\kappa_2 + \rho_{i3}\kappa_3} = \frac{(\rho_{i1} + \rho_{i2})\eta_{i12} + \rho_{i3}\kappa_{i3}\eta_3}{(\rho_{i1} + \rho_{i2}) + \rho_{i3}\kappa_{i3}}$$

where

$$\eta_{i12} = \frac{\rho_{i1}\eta_1 + \rho_{i2}\kappa_2\eta_2}{\rho_{i1} + \rho_{i2}\kappa_2} = \frac{\rho_{i1}}{\rho_{i1} + \kappa_2\rho_{i2}}\eta_1 + \left(1 - \frac{\rho_{i1}}{\rho_{i1} + \kappa_2\rho_{i2}}\right)\eta_2 \text{ and } \kappa_{i3} = \frac{\rho_{i1} + \rho_{i2}}{\rho_{i1} + \kappa_2\rho_{i2}}\kappa_3.$$

Thus the combined cell type eQTL parameter is now weighted between the first two cell type eQTLs where the weights are functions of cell type proportion and κ_2. Also, for the TReC model's offset term, we have

$$\begin{aligned}
\log\left(\sum_q \rho_{iq}\kappa_q\right) &= \log\left(\rho_{i1} + \rho_{i2}\kappa_2 + \rho_{i3}\kappa_3\right) = \log\left(\frac{\rho_{i1} + \rho_{i2}\kappa_2}{\rho_{i1} + \rho_{i2}}(\rho_{i1} + \rho_{i2}) + \rho_{i3}\kappa_3\right) \\
&= \log\left(\frac{\rho_{i1} + \rho_{i2}\kappa_2}{\rho_{i1} + \rho_{i2}}\left[\rho_{i1} + \rho_{i2} + \frac{\rho_{i1} + \rho_{i2}}{\rho_{i1} + \rho_{i2}\kappa_2}\rho_{i3}\kappa_3\right]\right) \\
&= \log\left(\frac{\rho_{i1} + \rho_{i2}\kappa_2}{\rho_{i1} + \rho_{i2}}\right) + \log\left(\rho_{i1} + \rho_{i2} + \rho_{i3}\kappa_{i3}\right)
\end{aligned}$$

Thus, if we collapse a subset of cell types and proceed with optimization, this assumes that these sample-specific terms η_{i12}, κ_{i3}, and $\log\left(\frac{\rho_{i1} + \rho_{i2}\kappa_2}{\rho_{i1} + \rho_{i2}}\right)$ are constant parameters for all i. These sample-specific terms can potentially explain a source of variability in TReC that is lost through collapsing cell types. Given our framework, this may lead to Type I Error inflation which we can observe through SNP permutation analyses. However, if a subset of cell types, denoted Q_s, present at very similar proportions across all samples (i.e. $\rho_{iq} \approx \rho_{jq}$ for samples i, j and $q \in Q_s$), then collapsing cell types can be justified and recommended since cell types with narrow spread in proportion can lead to unstable κ_q, η_q, and α_q estimates.

Supplementary Note 3: Multiple Testing and FDR

Just as in existing eQTL mapping methods, the issue of multiple testing arises for a gene's tested SNPs. The linkage disequilibrium (LD) of nearby SNPs leads to redundancy and so, similar to Sun (2012) [14] and Hu et al. [15], we propose selecting the most significant SNP per gene and cell type after accounting for the multiple testing. But unlike the aforementioned approaches, CSeQTL does not scale well with using SNP permutation to approximate a gene's minimum p-value null distribution from the added computation of cell type-specific eQTLs and reference expression fold changes. For the gth gene, suppose there are S_g corresponding SNPs. If S_g tests along a gene were independent one could simply apply Bonferroni adjustment to the eQTL significance testing p-values. However due to LD, we risk a loss in power. Instead, we chose to employ the geoP approach [16] for approximating the number of independent tests among S_g SNPs to approximate the permutation p-value. Let $\tilde{S}_g$ denote the number of independent tests from geoP and let p_{gs} denote the sth SNP's nominal p-value tested for a specific analysis (bulk or cell type-specific). Let p_g denote the gth gene's minimum p-value SNP approximated permutation p-value and defined as $p_g = \min\left(1, \min_s(p_{gs})\tilde{S}_g\right)$.

With regard to assessing the number of genes with eQTLs, we control for the false discovery rate (FDR) using the approach by Storey and Tibshirani [17] with a small modification. This is due to the observation that many permutation p-values equal 1.0. First, FDR is defined as

$$\text{FDR}(p_c) = \frac{\pi_0 p_c N_G}{\sum_{g=1}^{G} 1\{p_g \le p_c\}},$$

where π_0 is the expected proportion of true null hypotheses, p_c is the significance p-value cutoff, $1\{A\} = 1$ if A is true and $1\{A\} = 0$ otherwise, and N_G is the number of genes tested. Indexed by λ, the parameter π_0 is typically estimated as

$$\widehat{\pi}_0(\lambda) = \frac{1}{N_G} \sum_{g=1}^{G} \frac{1\{p_g \geq \lambda\}}{1-\lambda},$$

where $0 < \lambda < 1$. Our modification assumes the observed p_g's equal to 1.0 are actually continuous uniformly distributed between 0.0 and 1.0 and thus we have

$$\widehat{\pi}_0(\lambda) = \frac{1}{N_G} \sum_{g=1}^{G} \left[\frac{1\{1 > p_g \geq \lambda\}}{1-\lambda} + 1\{p_g = 1\} \right],$$

where we chose to set $\lambda = 0.5$. We can search over a grid of values p_c to achieve the desired FDR (such as 0.01, 0.05, 0.10). The q-value is then calculated as

$$\text{q-value}(p_g) = \min_{p_c \geq p_g} \text{FDR}(p_c).$$

Supplementary Note 4: Data Preprocessing

We assessed data collected from the CommonMind Consortium (CMC) [18, 19], BLUEPRINT [20] for its purified blood cell types, Genotype Tissue Expression's (GTEx) Whole Blood (WB) and Brain Frontal Cortex (BFC).

Among CMC samples, we obtained 606 RNA-seq bam files and corresponding genotypes, from SNP6 microarray, on control and schizophrenic (SCZ) samples using the Synapse platform. For CMC, starting with 752,269 genotyped SNPs by Illumina Infinium HumanOmniExpressExome 8v1.1b chip, we arrived at 5,792,709 imputed and phased SNPs across autosomes using 1,000 Genomes Phase 3 reference panels after phasing with SHAPEIT v2.837 [21], imputation of variants in 5 Mb segments with IMPUTE2 v2.3.2 [22], retaining loci with info metric >0.3, minor allele frequency >0.05, and SNPs with allelic dosages >0.8, and lifting coordinates from GRCh37 to GRCh38.

For the BLUEPRINT cohort, we used the European Genome Archive's pyega3 client to download phased SNPs derived from whole genome sequencing (EGAD00001002663) and three purified cell types of RNA-seq bam files (EGAD00001002671, EGAD00001002674, EGAD00001002675) corresponding to CD4+ alpha-beta T cell (n=212), CD14+/CD16- monocytes (n=197), and mature neutrophil (n=205), respectively. BLUEPRINT's VCF contained phased GRCh37 loci from whole genome sequencing that we lifted over to GRCh38 resulting in 4,851,911 successfully mapped and phased SNPs.

For GTEx WB and BFC cohorts, preprocessed phased variants from whole genome sequencing were downloaded from `https://anvil.terra.bio/#workspaces/anvil-datastorage/AnVIL_GTEx_V8_hg38`. GTEx's VCF provided 5,938,874 phased SNPs mapped to GRCh38. Preprocessed GRCh38-aligned RNA-seq bam files were obtained and processed on Google Cloud to obtain TReC and ASReC per gene through the AnVIL platform, using the pipeline from the GitHub repository, gtex_AnVIL: `https://github.com/Sun-lab/gtex_AnVIL` [16].

We have reprocessed the RNA-seq bam files from CMC and BLUEPRINT following GTEx's pipeline, available at `https://github.com/broadinstitute/gtex-pipeline/tree/master/rnaseq`, and contained in Figure 10. RNA-seq bam files were converted from bam to fastq with Picard v2.21.7 [23]. Samples were aligned using reference genome GRCh38 excluding ALT, HLA, and Decoy contigs but including ERCC spike-ins. Gene models were based on GENCODE's v26 GRCh38 with ERCC spike-in. The STAR index was constructed using the aforementioned reference genome and gene models. Reads were aligned using STAR v2.5.3a with input parameters provided by GTEx's template. Bam files were subsequently sorted by coordinate and indexed with Samtools v1.8 and duplicated reads were marked by Picard. With CMC's imputed SNPs and WGS SNPs for BLUEPRINT, WB, and BFC, reads were next sorted by read name. Then,

bam files were passed into asSeq [24] v0.99.501 to obtain TReC and ASReC for haplotype 1 or haplotype 2. In addition, some RNA-seq reads that harbor more than one heterozygous SNPs are not compatible with either haplotype. We refer to those as haplotype-ambiguous reads.

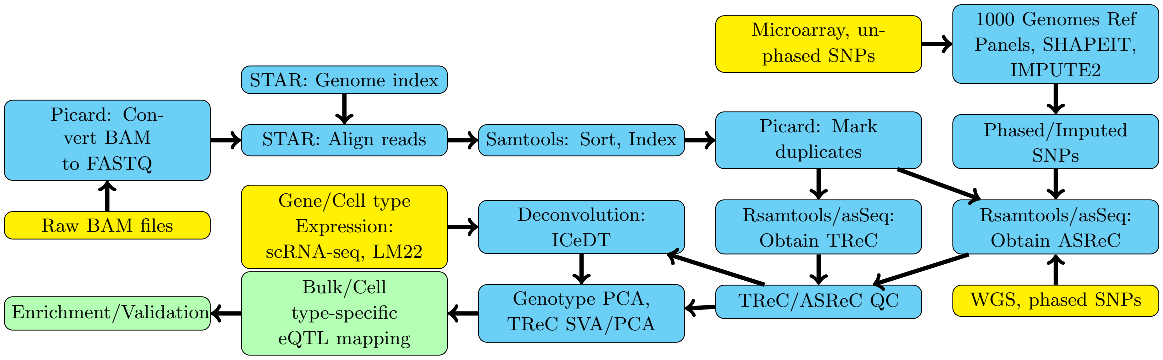

Supplementary Figure 10: **Analysis Workflow.** Yellow nodes correspond to initial input data files. Blue nodes correspond to intermediate workflow steps. Green nodes correspond to our contributed analyses.

Supplementary Note 5: Quality Control, Filtering, ASReC adjustment

We started by plotting samples' total TReC versus total ASReC summed across genes to identify potential outliers. The exact threshold varied across studies as described next. In addition, we also examined the proportion of allele-specific reads per sample (i.e., total ASReC divided by total TReC) and filtered out samples with extreme proportions with study-specific thresholds.

Next we performed QC for allele-specific expression. If a gene had more than 10 allele-specific reads in a sample, we calculated the fraction of allele-specific reads mapped to the first haplotype, denoted by h_{gi}. If $|h_{gi} - 0.5| > 0.4$, we considered it as an extreme ASReC fraction. At the gene level, we examined the total ASReC across all samples as well as the total number of haplotype-ambiguous reads, if the fraction of haplotype-ambiguous reads was greater than 5%, we set a gene's ASReC to zero for all samples. Also, if the proportion of samples with extreme ASReC fractions was greater than 20% and mean proportion of haplotype-ambiguous reads was greater than 1%, we set that gene's ASReC to zero in all samples. Finally, for one gene and one sample, if the proportion of haplotype-ambiguous reads was greater than 10% and the number of allele-specific reads was greater than 10, we set its ASReC to zero.

From CMC, we selected the samples with both RNA-seq and genotype data, labeled as control or SCZ, and were not labeled as outlier by Fromer et al. [18]. Among the selected samples, we calculated the proportion of allele-specific reads per sample and excluded one sample with a low proportion less than 0.04. Next, we removed one sample with the proportion of haplotype-ambiguous reads being larger than 0.4%. These filtering criteria resulted in 537 samples (283 controls, 254 SCZ).

For BLUEPRINT, there were 548 samples with both RNA-seq and genotype data: 176 monocyte, 211 CD4+ T cell, and 161 neutrophil. With each purified cell type cohort having a mixture of paired and single-end read sequencing, we selected single-end sequencing samples for monocyte (n=173) and neutrophil (n=154) cohorts and paired-end sequencing samples for CD4+ T cell (n=208). None of the remaining samples appeared to deviate in terms of ASReC vs. TReC. When we inspected the proportion of haplotype-ambiguous reads, four T cell samples were excluded after applying a 0.25% cutoff. These criteria resulted in 531 samples (173 monocytes, 204 CD4+ T cells, 154 neutrophils).

For GTEx's BFC, there were 175 samples with both RNA-seq and genotype data. One sample appeared to have an extremely high TReC across genes and was excluded. When we compared ASReC vs TReC per

sample, there were 17 samples with the proportion of allele-specific reads larger than 5.5%. Unlike the above filtering procedure, we retained these samples but set their ASReC to zero to maintain a similar sample size with the recent GTEx eQTL analysis [25] resulting in 174 samples.

For WB, GTEx contained 670 samples with available RNA-seq and genotype data. None of the samples were considered outliers based on TReC across genes. When we compared ASReC versus TReC, in 68 samples the proportions of allele-specific reads were greater than 7%. Similar to BFC, rather than excluding these samples, we retained them but set their ASReC to zero to maintain the sample sizes in the GTEx analysis [25].

Cohort	Counts				SNPs per Gene		
	Samples	Genes	SNPs	Gene+SNP pairs	Q25	Q50	Q75
CommonMind Consortium - Control	275	15,334	5,792,709	32,523,198	1,661	2,045	2,462
CommonMind Consortium - SCZ	250	15,275	5,792,709	32,186,224	1,645	2,036	2,450
GTEx Brain	174	15,400	5,938,874	33,326,549	1,665	2,093	2,546
BLUEPRINT - CD4T	167	13,339	4,851,911	22,532,356	1,304	1,672	2,035
BLUEPRINT - Monocyte	173	13,624	4,851,911	23,101,424	1,315	1,680	2,034
BLUEPRINT - Neutrophil	151	10,769	4,851,911	18,044,122	1,298	1,662	2,016
GTEx Blood	670	12,022	5,938,874	26,426,763	1,697	2,124	2,581

Supplementary Table 1: **Summary of sample size, the number of genes, SNPs, local gene-SNP pairs, and the number of local SNPs per gene.** Q25, Q50, and Q75 indicate 1st quartile, median and 3rd quartile.

Supplementary Note 6: Real Data Analyses

6.1 Covariates

For bulk and cell type-specific models with CMC, covariates included log transformed 75th percentile of TReC (library size), sex, institution, binned library cluster obtained from the supplemental materials of Fromer et al. [18], age at death, postmortem interval, RNA integrity number, RNA integrity number squared, and the top five genotype principal components.

For the BLUEPRINT dataset, covariates included library size, sex, donor age, submission year, % unique reads, % coding bases, % UTR bases, the top five genotype principal components, and the top sixteen residual TReC principal components per purified cell type.

For the GTEx WB dataset, covariates included library size, sex, sequencing protocol, sequencing platform, sequencing center, RNA integrity number, RNA integrity number squared, donor age, base mismatch rate, % reads mapped within exons, number of mapped read pairs, mean fragment length, % intronic reads, % rRNA reads, number of genes detected with ≥ 5 exon reads, end 1 mismatch rate (fraction of end 1 bases not matching the reference), fragment length standard deviation, intergenic rate, number of rRNA reads, number of reads labeled failed by sequencer, ratio of exon reads to total reads, end 2 mismatch rate (fraction of end 2 bases not matching the reference), end 2 % sense (percentage of intragenic end 2 reads sequenced in the sense direction), and the top ten genotype principal components.

For the GTEx brain dataset, covariates included library size, sex, sequencing protocol, % rRNA reads, end 2 mapping rate, intergenic rate, end 2 % sense, binned year of nucleic acid isolation batch, RNA integrity number, fragment length standard deviation, total filtered reads, number of alternate alignments, number of reads spanning an exon-exon boundary, end 1 % sense, and the top three genotype principal components. GTEx dataset variables and data dictionary were obtained at **https://www.gtexportal.org/home/datasets**.

For CMC and GTEx datasets, we also calculated PCs based on log-transformed TReC data. We first fit a linear model of log transformed TReC data on all the aforementioned covariates, and then calculated PCs (or surrogate variables) using the residuals of the linear model. In addition, we also considered another linear model that included aforementioned covariates as well as cell type proportions, and then calculated PCs (or surrogate variables) using the residuals of this larger model. Therefore, these two sets of PCs capture the latent batch effects that were ignorant or orthogonal to cell type proportion. For CMC, we used the top twelve surrogate variables calculated independent of disease status with SVA [26]. For GTEx WB and brain samples, latent effects were captured by the top 20 PCs and top 16 PCs from residualized TReC, respectively.

6.2 Commonmind Consortium

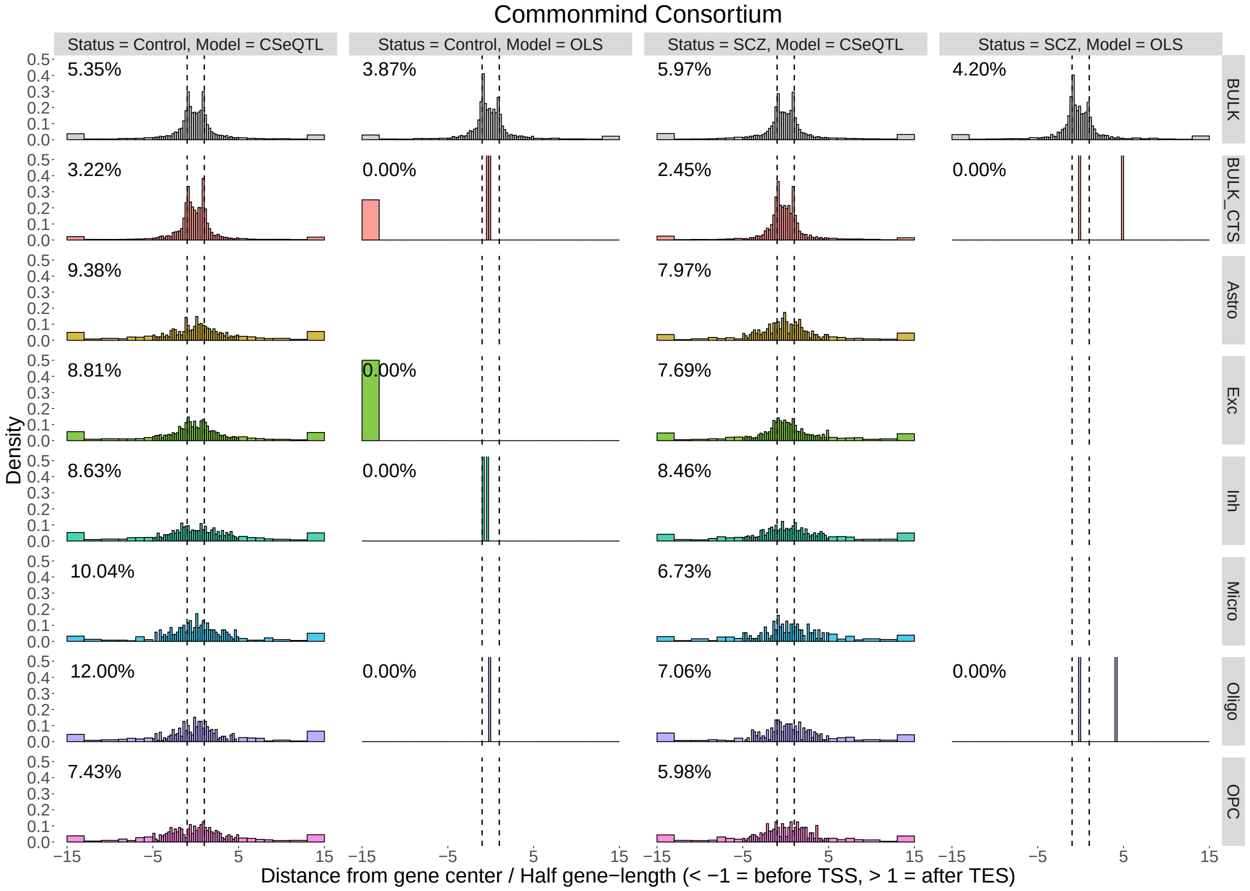

Supplementary Figure 11: **The distribution of eQTLs from the CMC study.** The distribution of normalized distance between each eGene (the center of its gene body) and its minimum p-value SNP. The raw distance (base pairs) were normalized by dividing by half of the gene size. Therefore $[-1, 1]$ was the range of gene body. Percentages of eGene for which such distances were beyond 15 units were provided at the upper-left corner of each facet plot. BULK refers to the eGenes detected by bulk eQTL mapping while BULK_CTS refers to all bulk eGenes identified as cell type-specific eGenes in at least one cell type.

TRIM	COHORT	MODEL	CELLTYPE	Perm PVAL	ERROR	eGene	OL_eGene	eQTL	OL_eQTL
FALSE	Control	CSeQTL	Bulk	2.20e-02	0.07	8,116	-	835,196	-
			Astro	3.22e-04	0.05	716	70.17%	7,606	73.49%
			Exc	1.76e-03	0.05	2,511	75.23%	65,321	81.91%
			Inh	5.12e-04	0.05	1,133	61.61%	11,125	34.88%
			Micro	1.66e-04	0.05	264	68.94%	9,460	93.50%
			Oligo	3.15e-04	0.05	495	71.92%	5,525	78.75%
			OPC	2.01e-04	0.04	392	60.20%	3,310	60.00%
		OLS	Bulk	3.28e-03	0.05	3,812	-	374,302	-
			Astro	1.58e-06	0.06	0	-	0	-
			Exc	7.05e-08	0.05	1	100.00%	1	100.00%
			Inh	5.85e-07	0.05	2	100.00%	9	100.00%
			Micro	1.20e-06	0.06	0	-	0	-
			Oligo	1.16e-06	0.05	2	100.00%	45	100.00%
			OPC	7.83e-07	0.06	0	-	0	-
	SCZ	CSeQTL	Bulk	1.78e-02	0.07	7,228	-	704,913	-
			Astro	3.16e-04	0.05	626	61.50%	6,373	78.94%
			Exc	1.48e-03	0.06	1,888	72.62%	51,864	89.80%
			Inh	4.44e-04	0.05	979	57.46%	8,059	18.33%
			Micro	1.25e-04	0.06	183	60.66%	1,894	74.76%
			Oligo	2.02e-04	0.05	300	62.33%	4,533	83.76%
			OPC	1.60e-04	0.04	334	57.66%	2,157	64.44%
		OLS	Bulk	2.17e-03	0.05	2,919	-	285,391	-
			Astro	5.20e-06	0.07	0	-	0	-
			Exc	2.57e-06	0.06	2	100.00%	63	100.00%
			Inh	5.77e-06	0.06	0	-	0	-
			Micro	3.04e-06	0.07	2	0.00%	16	0.00%
			Oligo	1.11e-06	0.06	3	66.67%	20	85.00%
			OPC	2.46e-05	0.06	0	-	0	-
TRUE	Control	CSeQTL	Bulk	1.90e-02	0.07	7,780	-	807,352	-
			Astro	3.20e-04	0.04	725	65.10%	7,281	69.32%
			Exc	1.50e-03	0.05	2,337	74.27%	57,499	80.70%
			Inh	5.48e-04	0.05	1,217	58.34%	10,893	24.22%
			Micro	1.65e-04	0.05	279	62.72%	9,241	93.15%
			Oligo	2.90e-04	0.05	475	70.11%	5,872	79.00%
			OPC	2.29e-04	0.04	444	57.88%	2,849	57.11%
		OLS	Bulk	3.14e-03	0.05	3,774	-	374,638	-
			Astro	1.73e-05	0.05	0	-	0	-
			Exc	7.05e-08	0.05	1	100.00%	1	100.00%
			Inh	4.10e-07	0.05	2	100.00%	7	100.00%
			Micro	4.71e-05	0.06	0	-	0	-
			Oligo	7.90e-07	0.05	1	100.00%	23	100.00%
			OPC	2.72e-05	0.06	0	-	0	-
	SCZ	CSeQTL	Bulk	1.53e-02	0.07	6,885	-	680,924	-
			Astro	2.56e-04	0.05	552	58.33%	6,197	78.59%
			Exc	1.21e-03	0.06	1,716	69.80%	49,403	84.32%
			Inh	4.49e-04	0.05	1,016	49.61%	9,423	13.76%
			Micro	1.37e-04	0.05	223	47.09%	3,007	84.87%
			Oligo	2.08e-04	0.05	326	61.04%	5,203	83.05%
			OPC	1.75e-04	0.04	351	53.85%	2,070	61.11%
		OLS	Bulk	2.12e-03	0.05	2,902	-	286,245	-
			Astro	1.12e-04	0.06	0	-	0	-
			Exc	4.82e-07	0.06	0	-	0	-
			Inh	8.75e-06	0.06	0	-	0	-
			Micro	1.67e-05	0.06	0	-	0	-
			Oligo	9.05e-08	0.05	2	100.00%	10	100.00%
			OPC	2.18e-05	0.06	0	-	0	-

Supplementary Table 2: **Commonmind Consortium analysis.** Perm PVAL is the permutation p-value cutoff for q-value 0.005. Type I Error (ERROR) corresponds to proportion of eQTLs identified at p-value 0.05 after permuting SNP genotype data. OL_eGene and OL_eQTL are the percent of ct-eGenes and ct-eQTLs that are discovered by bulk eQTL mapping.

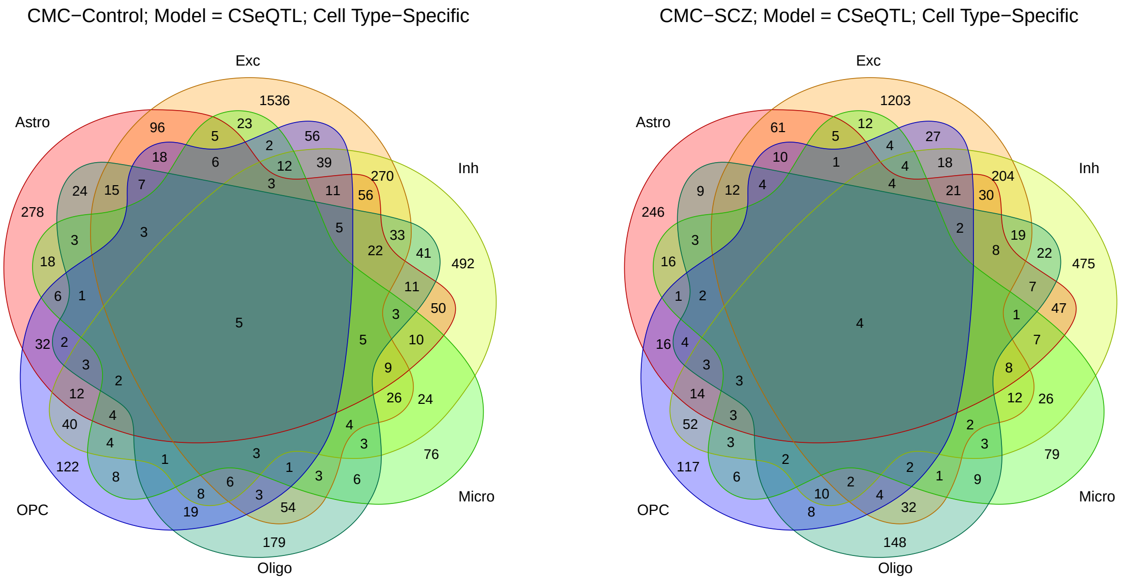

Supplementary Figure 12: **Overlapping eGenes among Commonmind Consortium control samples (left panel) and schizophrenia samples (right panel).**

6.3 GTEx Brain

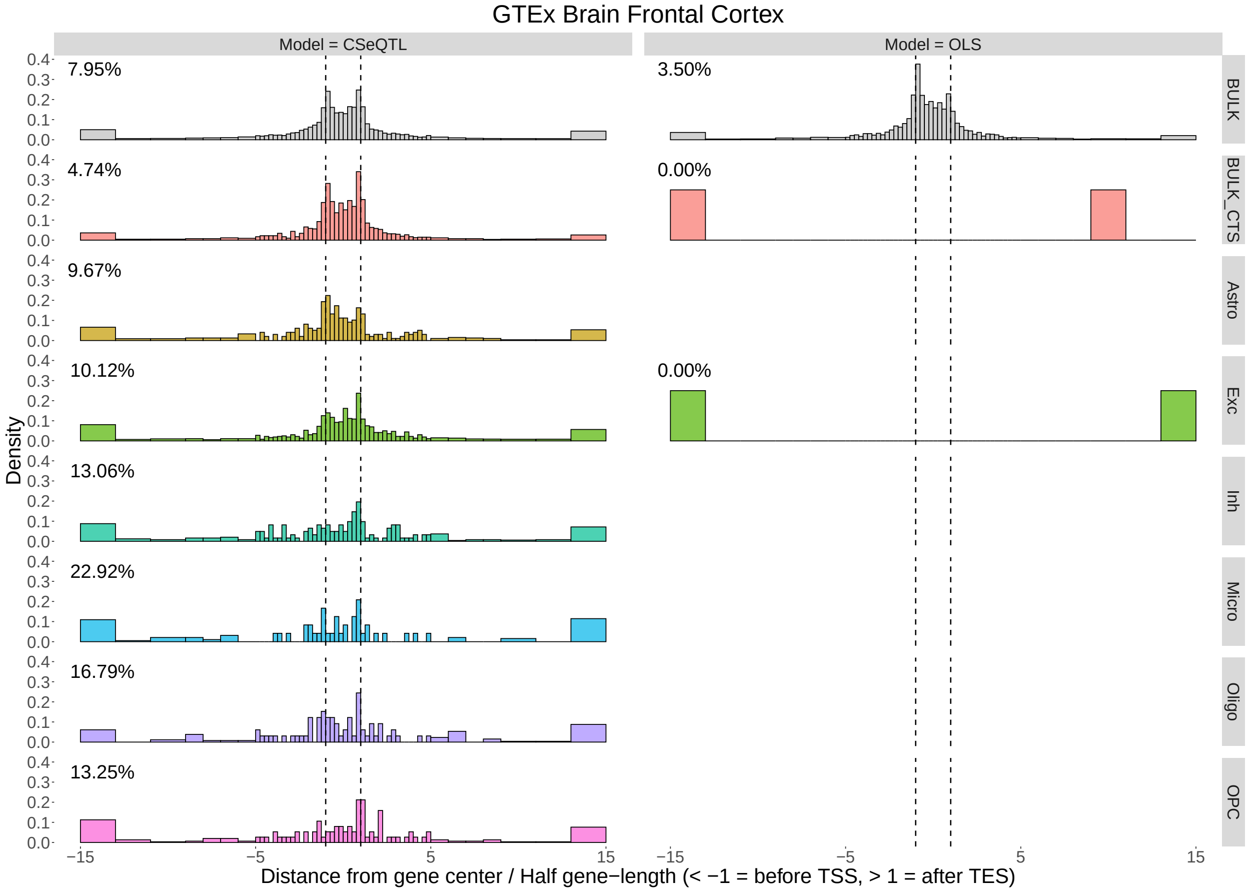

Supplementary Figure 13: **The distribution of eQTLs from the GTEx brain study.** The distribution of normalized distance between each eGene (the center of its gene body) and its minimum p-value SNP. The raw distance (base pairs) were normalized by dividing by half of the gene size. Therefore $[-1, 1]$ was the range of gene body. Percentages of eGene for which such distances were beyond 15 units were provided per facet plot. BULK refers to the eGenes detected by bulk eQTL mapping while BULK_CTS refers to all bulk eGenes identified as cell type-specific eGenes in at least one cell type.

TRIM	MODEL	CELLTYPE	Perm PVAL	ERROR	eGene	OL_eGene	eQTL	OL_eQTL
FALSE	CSeQTL	Bulk	3.24e-02	0.09	8,769	-	718,012	-
		Astro	6.40e-04	0.08	748	76.78%	13,551	76.84%
		Exc	2.06e-03	0.08	1,780	77.25%	44,562	84.39%
		Inh	1.69e-04	0.06	265	71.70%	1,981	58.35%
		Micro	7.76e-04	0.08	885	50.62%	15,318	10.77%
		Oligo	3.90e-04	0.09	576	60.87%	6,585	44.60%
		OPC	1.06e-04	0.06	200	69.00%	2,655	80.38%
	OLS	Bulk	1.80e-03	0.05	2,683	-	215,762	-
		Astro	2.20e-05	0.07	0	-	0	-
		Exc	4.86e-06	0.06	12	83.33%	101	97.03%
		Inh	9.04e-06	0.05	0	-	0	-
		Micro	1.09e-04	0.11	184	3.30%	1,332	0.00%
		Oligo	9.39e-07	0.07	3	-	0	-
		OPC	7.31e-07	0.07	0	-	0	-
TRUE	CSeQTL	Bulk	2.97e-02	0.08	8,362	-	667,554	-
		Astro	2.42e-04	0.06	393	79.90%	6,910	83.44%
		Exc	1.35e-03	0.07	1,433	77.60%	37,734	84.46%
		Inh	1.40e-04	0.06	245	70.49%	2,144	63.34%
		Micro	5.15e-05	0.06	96	66.32%	1,650	84.61%
		Oligo	6.55e-05	0.06	131	67.69%	1,698	78.62%
		OPC	7.29e-05	0.05	151	60.67%	2,090	82.73%
	OLS	Bulk	1.75e-03	0.05	2,626	-	207,562	-
		Astro	1.15e-04	0.04	0	-	0	-
		Exc	1.62e-06	0.05	2	100.00%	10	100.00%
		Inh	4.58e-05	0.05	0	-	0	-
		Micro	1.28e-05	0.04	0	-	0	-
		Oligo	1.25e-05	0.05	0	-	0	-
		OPC	6.78e-05	0.04	0	-	0	-

Supplementary Table 3: **GTEx Brain Frontal Cortex (BA9) analysis.** Perm PVAL is the permutation p-value cutoff for q-value 0.005. Type I Error (ERROR) corresponds to proportion of eQTLs identified at p-value 0.05 after permuting SNP genotype data. OL_eGene and OL_eQTL are the percent of ct-eGenes and ct-eQTLs that are discovered by bulk eQTL mapping.

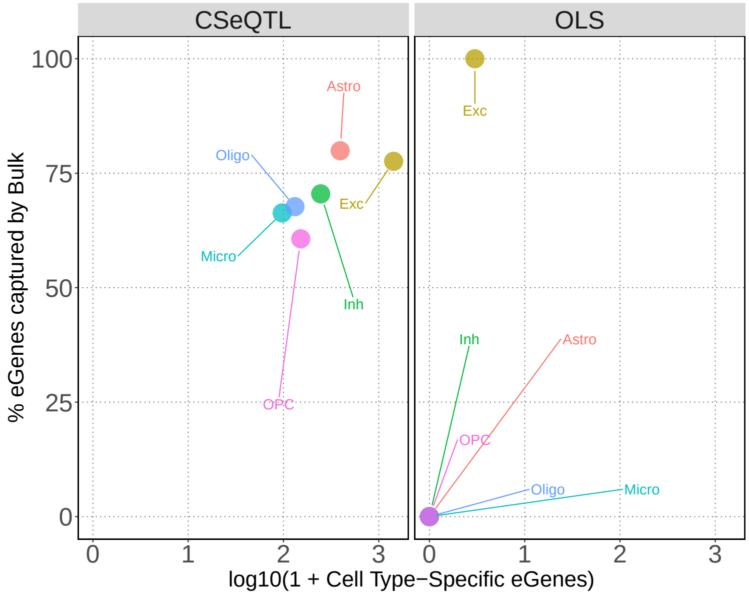

Supplementary Figure 14: **The number of eGenes from the GTEx brain study.** Comparison of the number of eGenes identified by CSeQTL or OLS method (X-axis) and what percentage overlap with the eGenes from bulk eQTL mapping.

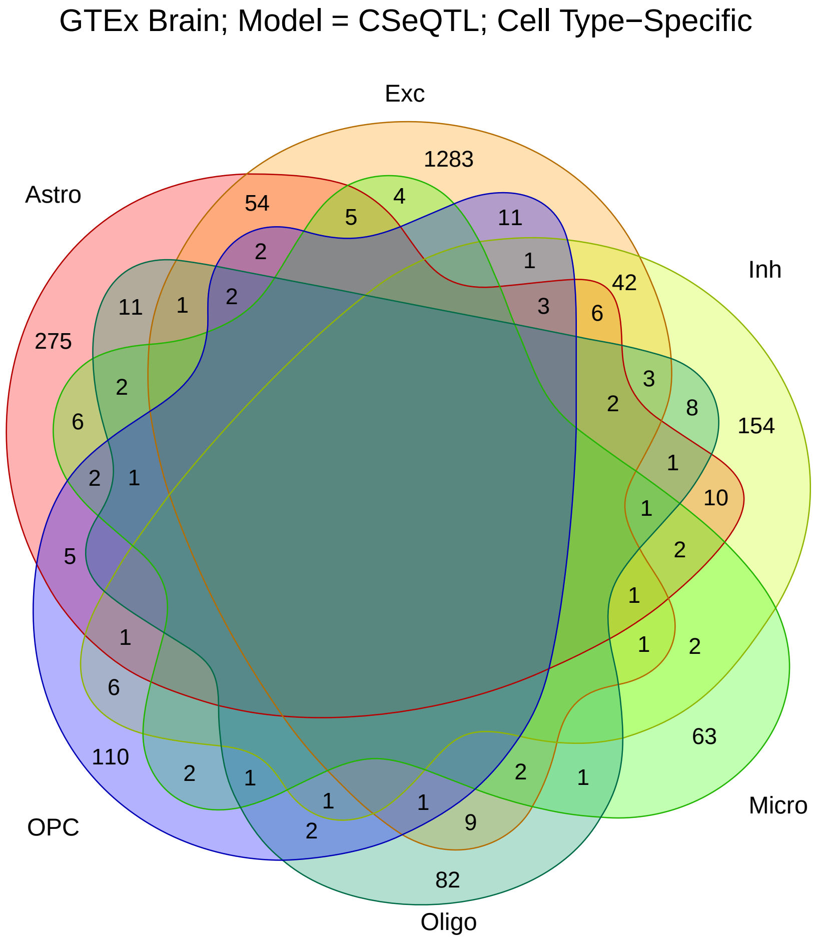

Supplementary Figure 15: **Overlapping eGenes among GTEx Brain samples.**

6.4 BLUEPRINT

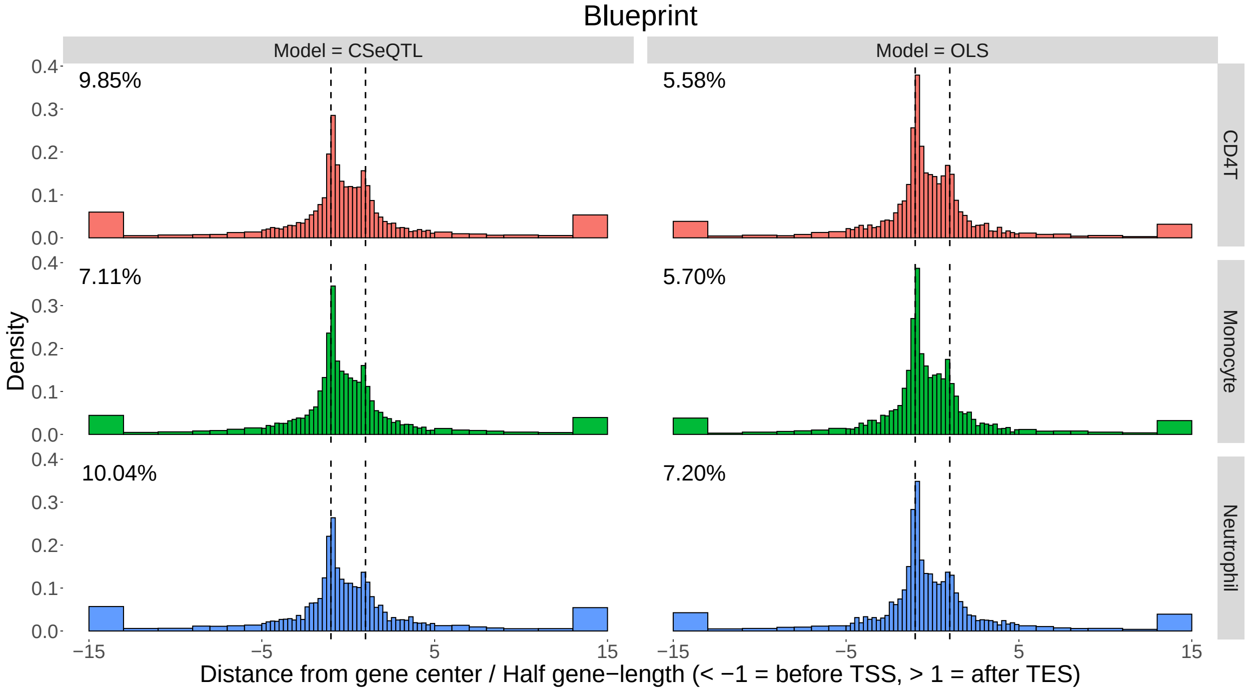

Supplementary Figure 16: **The distribution of eQTLs from the BLUEPRINT study.** The distribution of normalized distance between each eGene (the center of its gene body) and its minimum p-value SNP. The raw distance (base pairs) were normalized by dividing by half of the gene size. Therefore $[-1, 1]$ was the range of gene body. Percentages of eGene for which such distances were beyond 15 units were provided per facet plot.

TRIM	MODEL	CELLTYPE	Perm PVAL	ERROR	eGene	eQTL
FALSE	CSeQTL	CD4T	5.38e-02	0.08	9,450	983,572
	OLS		6.28e-03	0.05	5,268	459,610
	CSeQTL	Monocyte	1.36e-02	0.08	7,485	762,871
	OLS		6.57e-03	0.05	5,521	486,071
	CSeQTL	Neutrophil	2.29e-02	0.08	6,567	642,900
	OLS		5.62e-03	0.05	3,997	335,550
TRUE	CSeQTL	CD4T	5.10e-02	0.08	9,342	969,848
	OLS		6.21e-03	0.05	5,214	455,354
	CSeQTL	Monocyte	1.24e-02	0.07	7,304	750,037
	OLS		6.43e-03	0.05	5,470	481,877
	CSeQTL	Neutrophil	2.06e-02	0.08	6,402	628,963
	OLS		5.56e-03	0.05	3,972	332,108

Supplementary Table 4: **BLUEPRINT eQTL mapping results.** Perm PVAL is the permutation p-value cutoff for q-value 0.005. Type I Error (ERROR) corresponds to proportion of eQTLs identified at p-value 0.05 after permuting SNP genotype data.

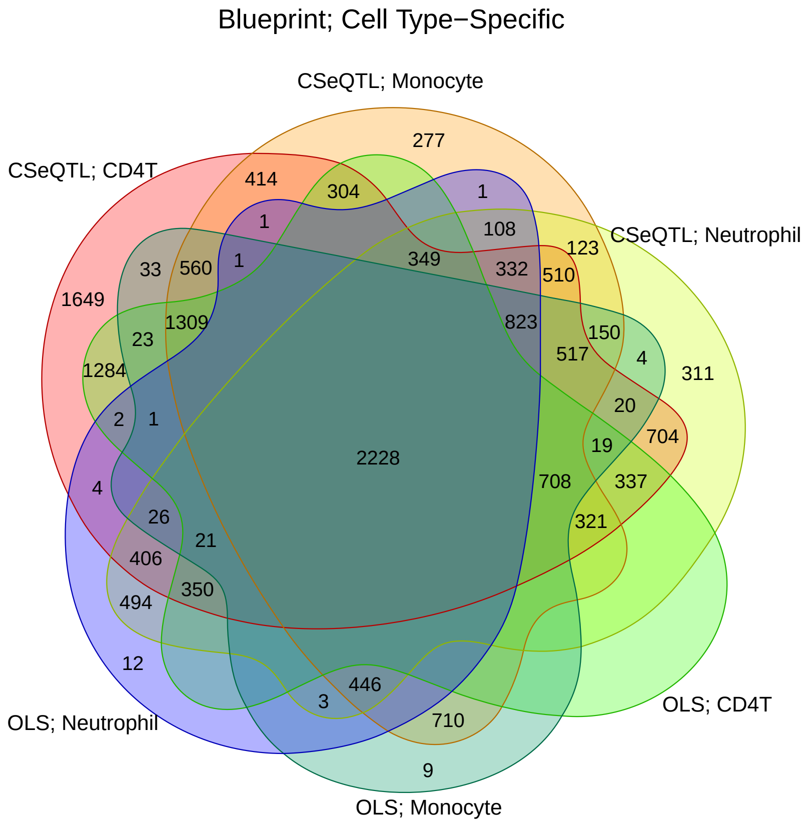

Supplementary Figure 17: **Overlapping eGenes among BLUEPRINT samples between cell types and models.**

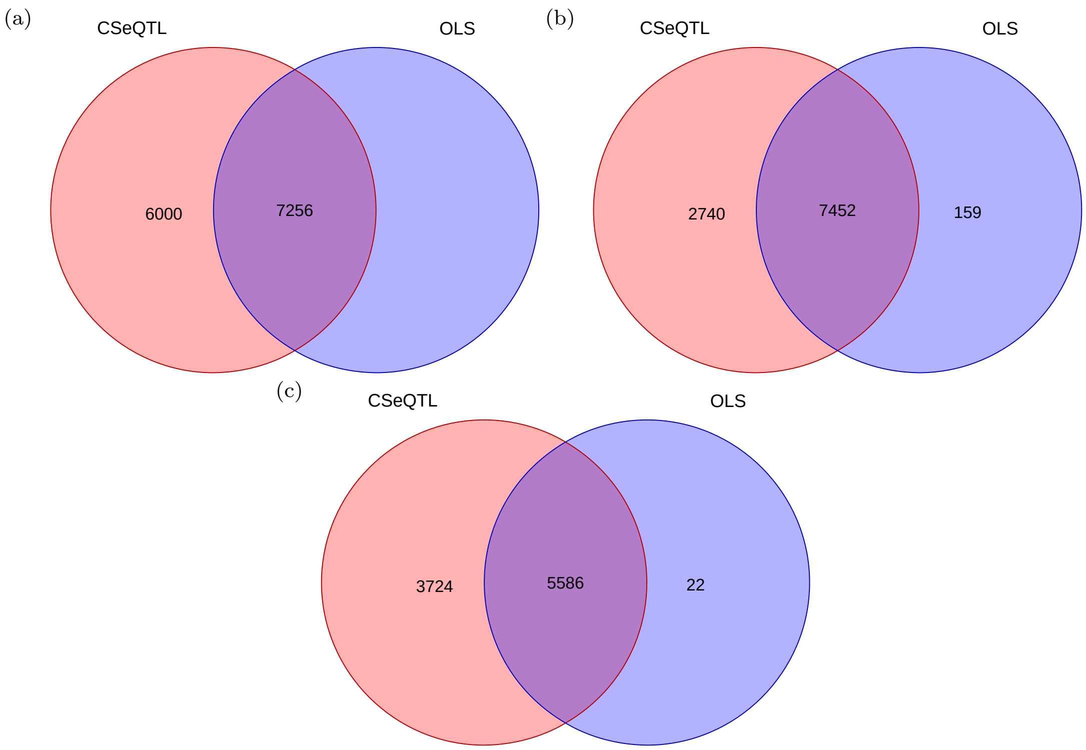

Supplementary Figure 18: **Shared and exclusive BLUEPRINT eGenes per cell type and by model.** (a) CD4+ T cells. (b) Monocytes. (c) Neutrophils.

6.5 GTEx Whole Blood

TRIM	MODEL	CELLTYPE	Perm PVAL	ERROR	eGene	OL_eGene	eQTL	OL_eQTL
FALSE	CSeQTL	Bulk	4.75e-02	0.07	9,079	-	1,422,635	-
		CD4T	1.26e-04	0.05	255	86.27%	4,229	75.05%
		CD8T	1.06e-04	0.05	172	90.70%	2,041	79.42%
		B_Cell	1.04e-04	0.05	130	85.38%	3,182	71.84%
		Monocyte	4.05e-04	0.06	438	89.73%	15,138	70.93%
		NK	3.72e-04	0.06	486	89.92%	14,193	82.58%
		Neutrophil	7.50e-03	0.07	4,371	95.29%	391,262	86.68%
		Mast_Eosinophil	1.88e-03	0.10	1,405	85.05%	37,853	64.58%
	OLS	Bulk	8.37e-03	0.05	5,521	-	699,125	-
		CD4T	5.05e-05	0.08	91	46.07%	875	8.57%
		CD8T	1.43e-06	0.05	0	-	0	-
		B_Cell	4.90e-07	0.07	1	-	0	-
		Monocyte	4.93e-05	0.08	59	38.98%	553	34.00%
		NK	3.65e-06	0.06	0	-	0	-
		Neutrophil	1.29e-03	0.07	1,125	93.07%	51,090	97.00%
		Mast_Eosinophil	1.93e-04	0.09	160	48.75%	1,143	26.07%
TRUE	CSeQTL	Bulk	4.01e-02	0.06	8,822	-	1,364,160	-
		CD4T	1.15e-04	0.04	223	85.65%	3,809	76.53%
		CD8T	1.22e-04	0.04	202	83.66%	2,394	72.64%
		B_Cell	1.00e-04	0.04	145	90.28%	2,899	84.37%
		Monocyte	3.74e-04	0.06	437	90.85%	17,928	71.64%
		NK	2.72e-04	0.05	436	92.20%	14,173	83.03%
		Neutrophil	6.09e-03	0.07	4,097	95.46%	362,627	86.65%
		Mast_Eosinophil	5.20e-04	0.07	579	90.33%	17,228	82.57%
	OLS	Bulk	8.39e-03	0.05	5,586	-	702,162	-
		CD4T	2.97e-05	0.04	0	-	0	-
		CD8T	4.24e-05	0.05	0	-	0	-
		B_Cell	1.65e-05	0.05	0	-	0	-
		Monocyte	9.81e-10	0.06	1	-	0	-
		NK	3.00e-05	0.05	0	-	0	-
		Neutrophil	8.75e-04	0.06	1,014	96.94%	47,168	97.84%
		Mast_Eosinophil	2.61e-07	0.07	2	100.00%	1	100.00%

Supplementary Table 5: **GTEx Whole Blood analysis.** Perm PVAL is the permutation p-value cutoff for q-value 0.005. Type I Error (ERROR) corresponds to proportion of eQTLs identified at p-value 0.05 after permuting SNP genotype data. OL_eGene and OL_eQTL are the percent of ct-eGenes and ct-eQTLs that are discovered by bulk eQTL mapping.

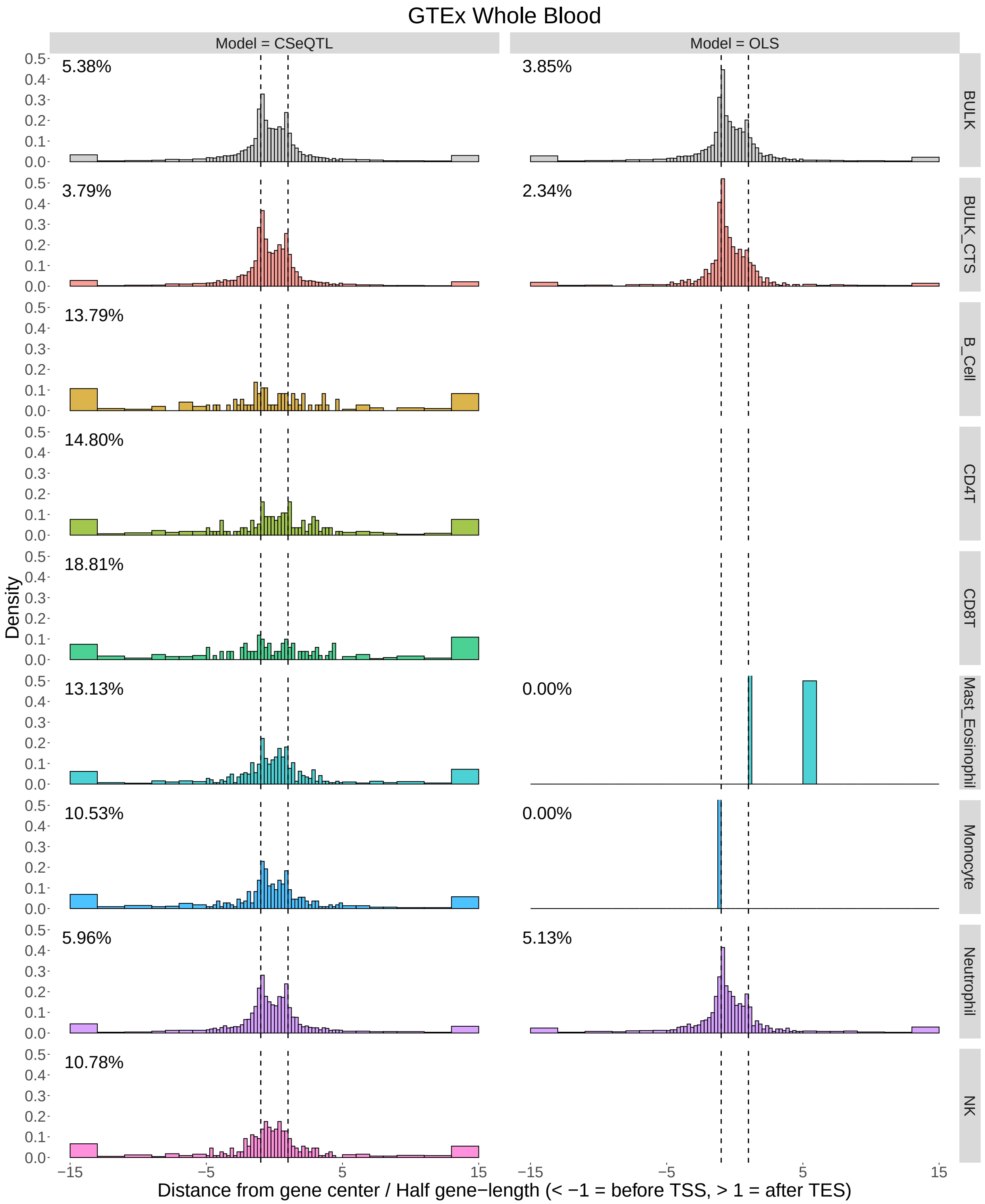

Supplementary Figure 19: **The distribution of eQTLs from the GTEx whole blood study.** The distribution of normalized distance between each eGene (the center of its gene body) and its minimum p-value SNP. The raw distance (base pairs) were normalized by dividing by half of the gene size. Therefore $[-1, 1]$ was the range of gene body. Percentages of eGene for which such distances were beyond 15 units were provided per facet plot. BULK refers to the eGenes detected by bulk eQTL mapping while BULK_CTS refers to all bulk eGenes identified as cell type-specific eGenes in at least one cell type.

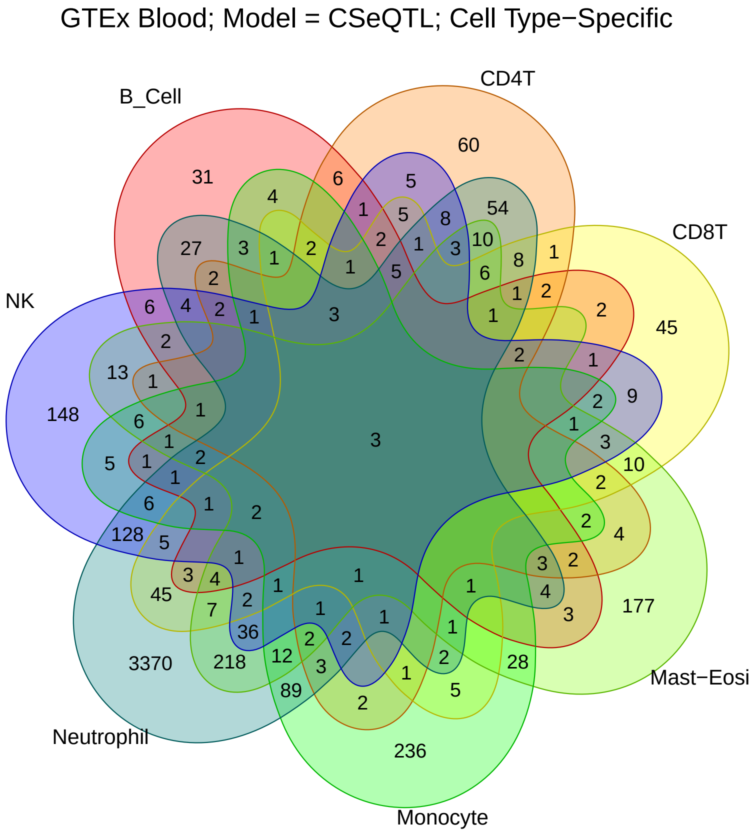

Supplementary Figure 20: **Overlapping eGenes among GTEx Blood samples.**

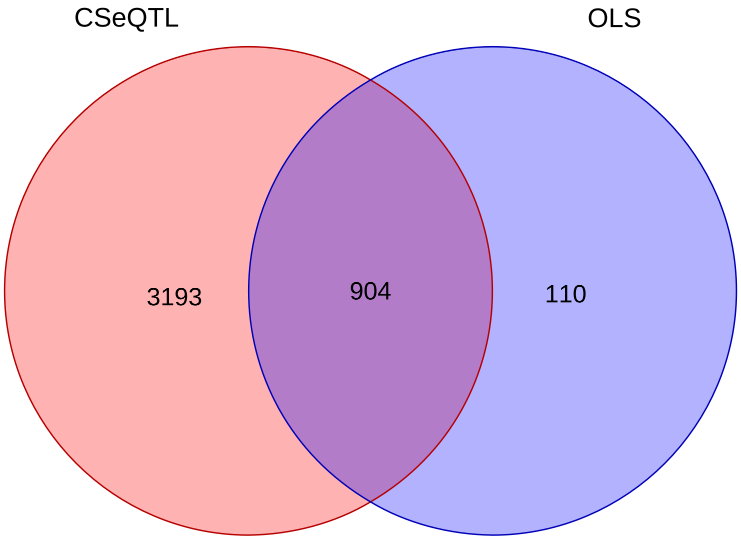

Supplementary Figure 21: **Overlapping eGenes among GTEx Blood samples for neutrophils between models.**

6.6 Validation

In addition to purified bulk RNA-seq data from BLUEPRINT, we also validated our method using sc-eQTLs from a scRNA-seq dataset of PBMC samples [27] and a snRNA-seq dataset of brain samples [28].

We obtained ct-eQTLs in 14 immune cell types reported by Yazar et al. [27]. They collected scRNA-seq data for almost 1,000 individuals (982 donors in total) with an average of 1,291 cells per donor, and named their cohort as OneK1K. Genotype data were collected from more than 5.3 million SNPs. ScRNA-seq data were generated using a pooled multiplexing strategy where each pool included cells from 12-14 donors. Cells were assigned to each donor using their genotype data. Gene expression data were normalized by SCTransform method [29] to remove the effect of read-depth, sequencing pool, as well as percentage of mitochondrial expression. The average expression of each gene and per person was calculated using the normalized counts. The covariates included were age, sex, six genotype PCs, and two PEER factors. They searched for local eQTLs within 1Mb of the gene, including gene body, by Spearman's rank correlation using residual expression. The FDR was evaluated for each chromosome separately, instead of a genome-wide search.

The other set of ct-eQTL results were derived by snRNA-seq data from brain samples. Bryois et al. [28] collected gene expression data of 6,940–14,595 genes and genotypes for 5.3 million SNPs in 144–192 individuals for eight major brain cell types: Excitatory neurons, Inhibitory neurons, Astrocytes, Microglia, Oligodendrocytes, Oligodendrocyte precursor cells (OPCs), Endothelial, and Pericytes. They searched for local eQTLs within 1Mb of the transcription start site (TSS). For each cell type and each individual, Bryois et al. [28] generated pseudo-bulk gene expression data by summing all counts for each gene. eQTL mapping was conducted by the fastQTL method that estimated the distribution of the minimum p-value per gene under the null (no eQTL), and then the authors calculated the permutation p-value for the minimum p-value per gene. Genes were filtered based on their average expression level (≥ 1 count per million) and proportion of ambient RNA ($< 10\%$). They used the following covariates: three first genotyping PCs, disease status (MS, AD, control or other), study (Roche_MS, Roche_AD, Columbia_AD, Mathys_AD and Zhou_AD) and 70 first expression PCs.

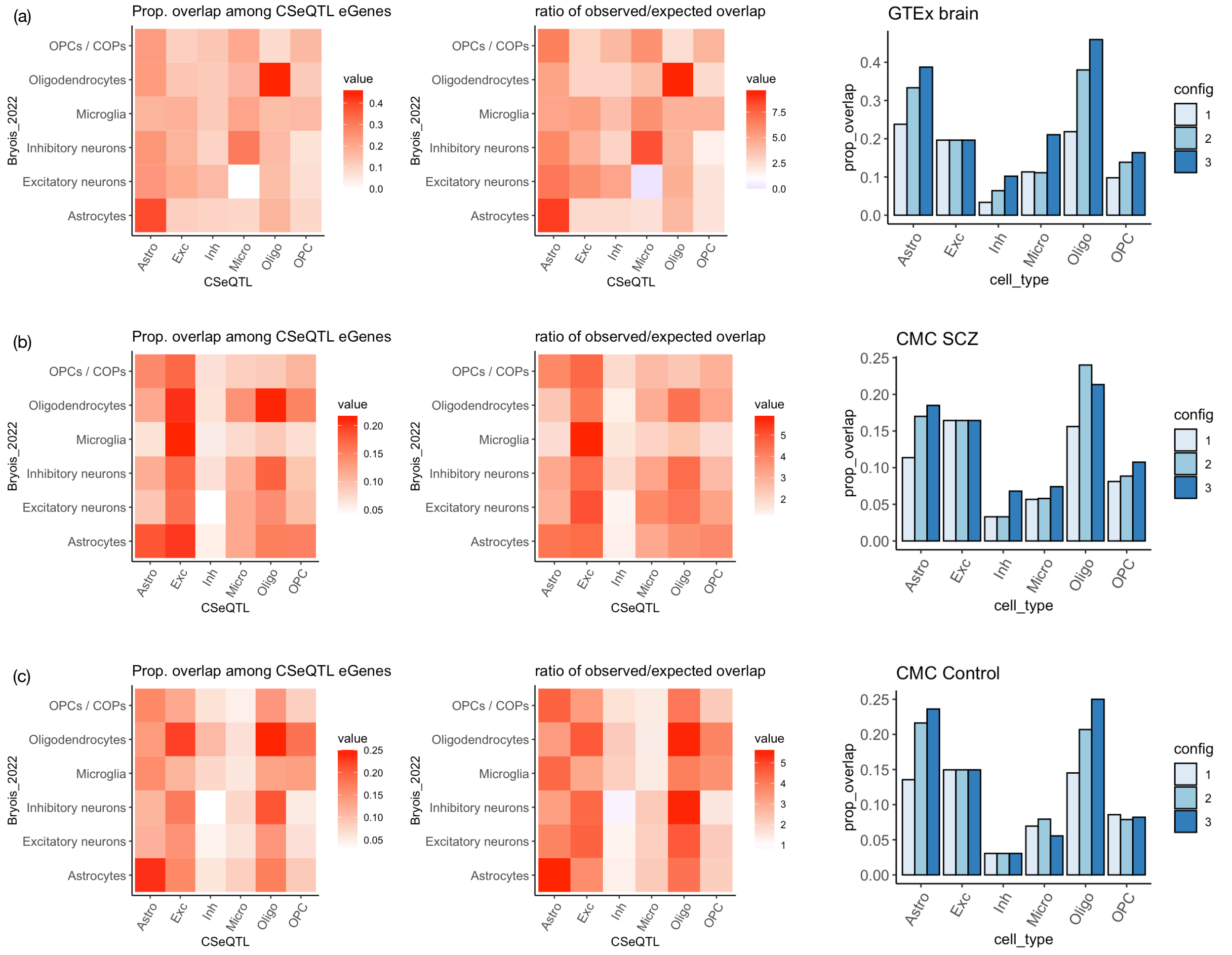

Supplementary Figure 22: **Validation of CSeQTL results brain bulk RNA-seq data** We compare the results by CSeQTL versus snRNA-seq data [28] in terms of proportion of overlap (among CSeQTL eGenes), the ratio of observed versus expected overlap and the trend of overlap with three configurations to define CSeQTL eGenes: (1) q-value < 0.005; (2) q-value < 0.005 and fold change ≥ 1.5; and (3) q-value < 0.001 and fold change ≥ 1.5. There are CSeQTL results for three brain bulk RNA-seq datasets: (a) GTEx brain, (b) CMC schizophrenia (SCZ), and (c) CMC controls.

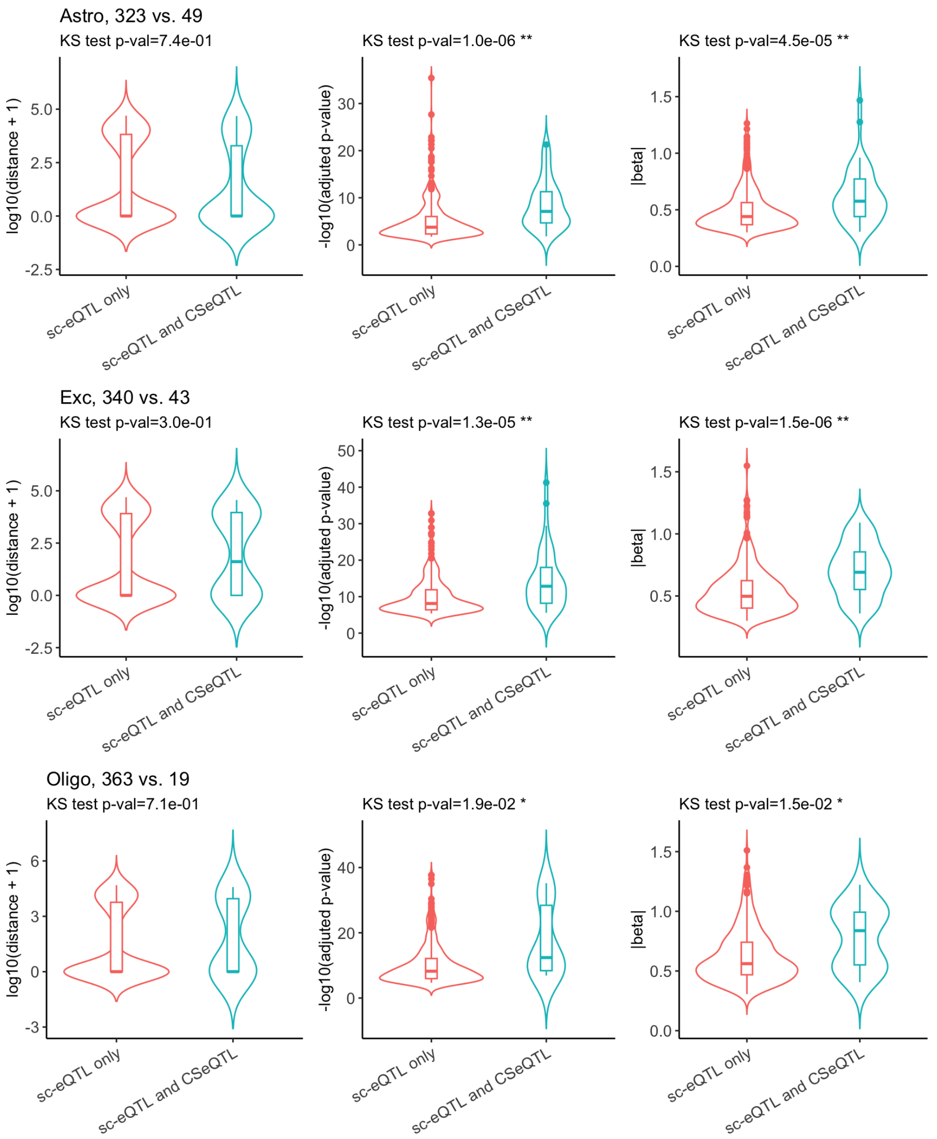

Supplementary Figure 23: **Characteristics of eGenes identified in sc-eQTL analysis.** Among the eGenes identified by scRNA-seq data [28], we compared those identified by sc-eQTL only versus those identified by both sc-eQTL and CSeQTL using GTEx brain data. Each row corresponds to a cell type. For example, in the first row, the title “Astro 323 vs. 49” indicates the comparison of 323 eGenes identified by scRNA-seq only versus 49 eGenes identified by both scRNA-seq and CSeQTL. The three columns correspond to three comparisons: distance between the eSNP with the smallest p-value to the corresponding eGene, which is 0 if the eSNP is within the eGene, adjusted p-value, and absolute value of effect size. For each comparison, we conducted two-sided Kolmogorov–Smirnov tests and significant results were labeled by ** (p-value < 0.01) or * ($0.01 \leq$ p-value < 0.05). For each boxplot, the box ranges from Q1 (the first quartile) to Q3 (the third quartile). The median is indicated by a line across the box. The whiskers extend from Q1 and Q3 to the most extreme data points within the 1.5 IQR of the box and IQR = Q3 − Q1.

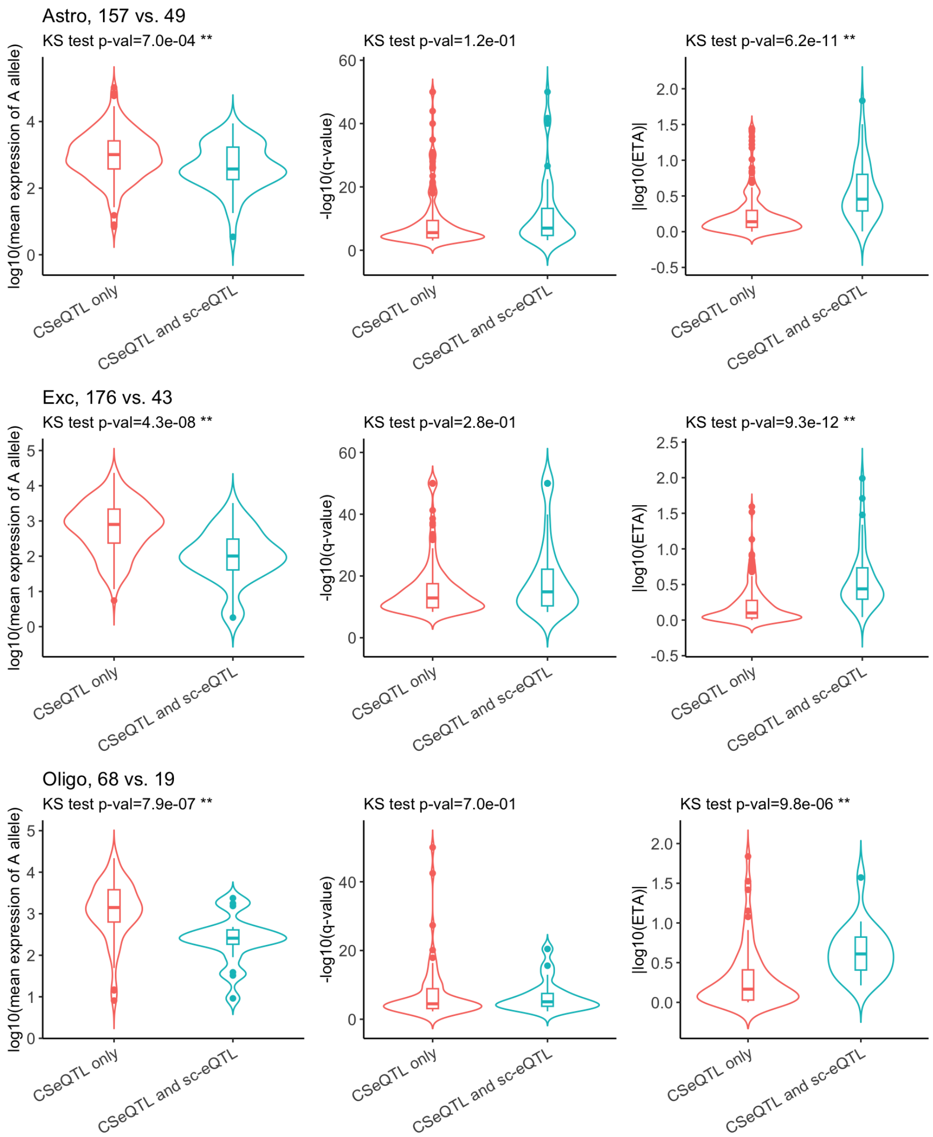

Supplementary Figure 24: **Characteristics of eGenes identified in CSeQTL analysis.** Among the eGenes identified by CSeQTL method using GTEx brain dat, we compared those identified by CSeQTL only versus those identified by both CSeQTL and sc-eQTL. Each row corresponds to a cell type. For example, in the first row, the title “Astro 157 vs. 49” indicates the comparison of 157 eGenes identified by CSeQTL only versus 49 eGenes identified by both CSeQTL and sc-eQTL. The three columns correspond to three comparisons: base-line expression of reference allele (A allele), q-value, and absolute value of effect size. Here effect size is quantified by ETA which is fold change. For each comparison, we conducted two-sided Kolmogorov–Smirnov tests and significant results were labeled by ** (p-value < 0.01) or * ($0.01 \leq$ p-value $<$ 0.05). For each boxplot, the box ranges from Q1 (the first quartile) to Q3 (the third quartile). The median is indicated by a line across the box. The whiskers extend from Q1 and Q3 to the most extreme data points within the 1.5 IQR of the box and IQR = Q3 − Q1.

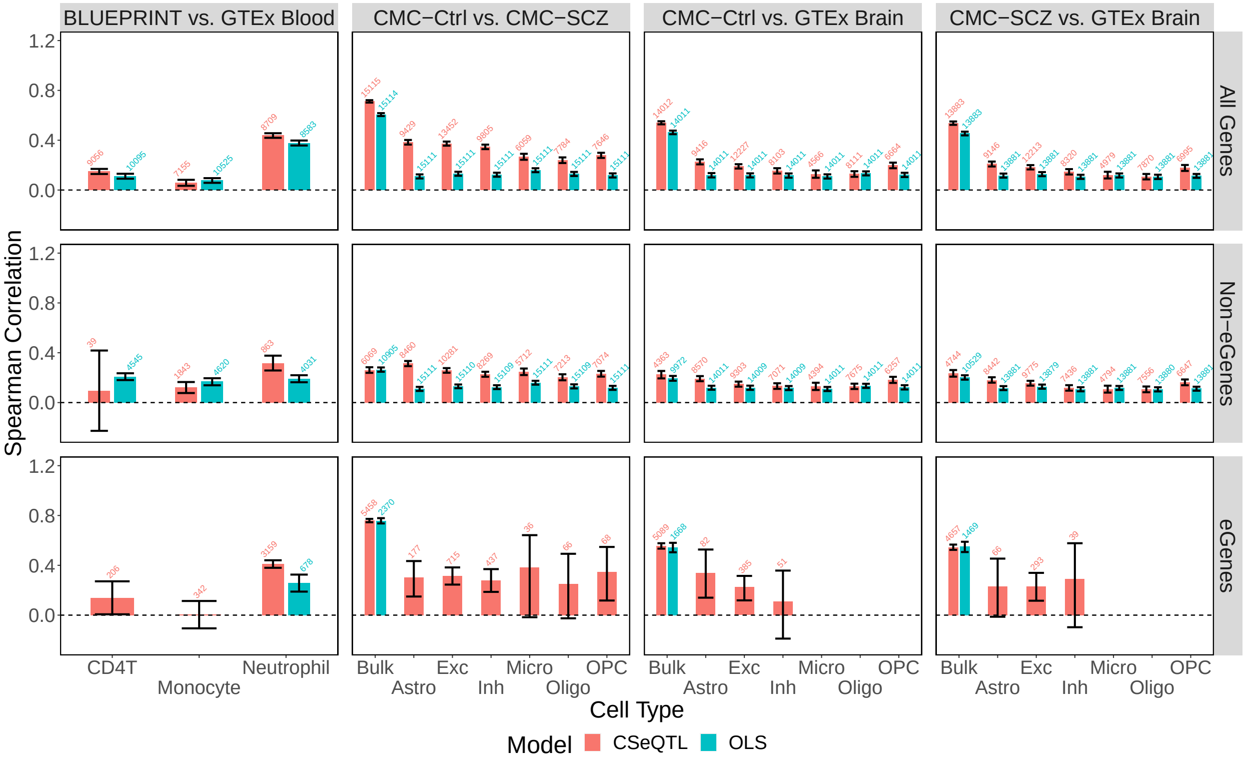

Supplementary Figure 25: **Comparing findings between data cohorts for shared cell types and between models.** The y-axis corresponds to the correlation of minimum eQTL p-value per gene between a pair of cohorts. 95% confidence intervals (with centers being the height of each barplot) are also plotted. For BLUEPRINT and GTEx Whole Blood, CD4 T cells, monocytes, and neutrophils are presented. For Commonmind Consortium and GTEx Brain Frontal Cortex, bulk and the six cell types are presented. Spearman correlation was calculated among all shared genes analyzed ("All Genes"), among shared eGenes ("eGenes"), and among all shared non-eGenes ("Non-eGenes"). The numbers above each bar plot are the number of genes used to derive the statistics and the confidence intervals. Plots are included for statistics calculated with at least 20 genes.

6.7 Consistency and enrichment

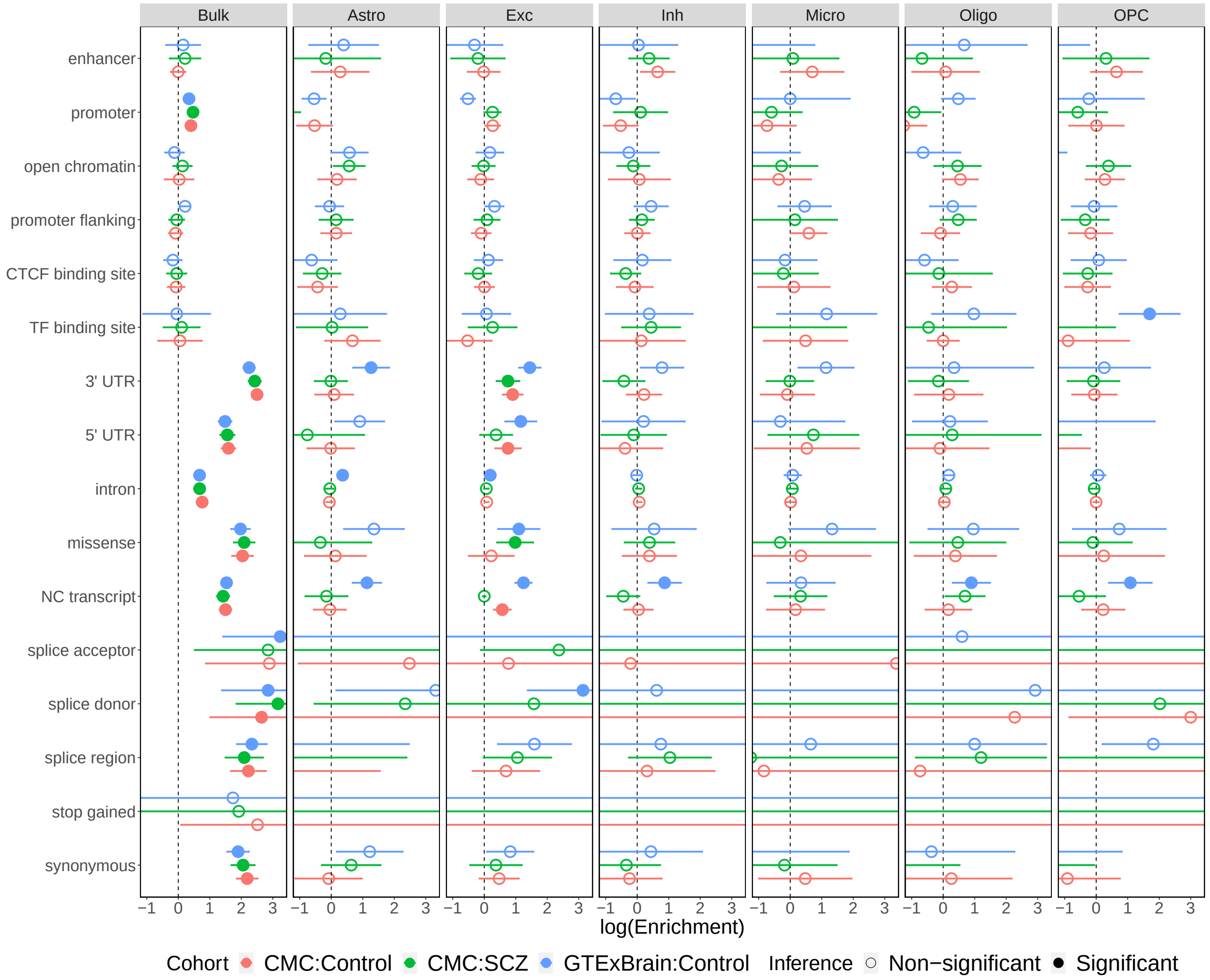

Supplementary Figure 26: **Functional enrichment for the eQTL results from the CMC cohorts and GTEx Brain samples.** Data are presented by point estimate of enrichment (each circle) and the 95% confidence interval (lines centered at point estimates). Nominal p-values are Bonferroni corrected to account for multiple comparison. Filled circles correspond to the ones with lower bound of confidence intervals larger than zero and adjusted p-values < 0.05. The enrichment estimates and their 95% confidence intervals were obtained by Torus with $n = 15,334$ genes.

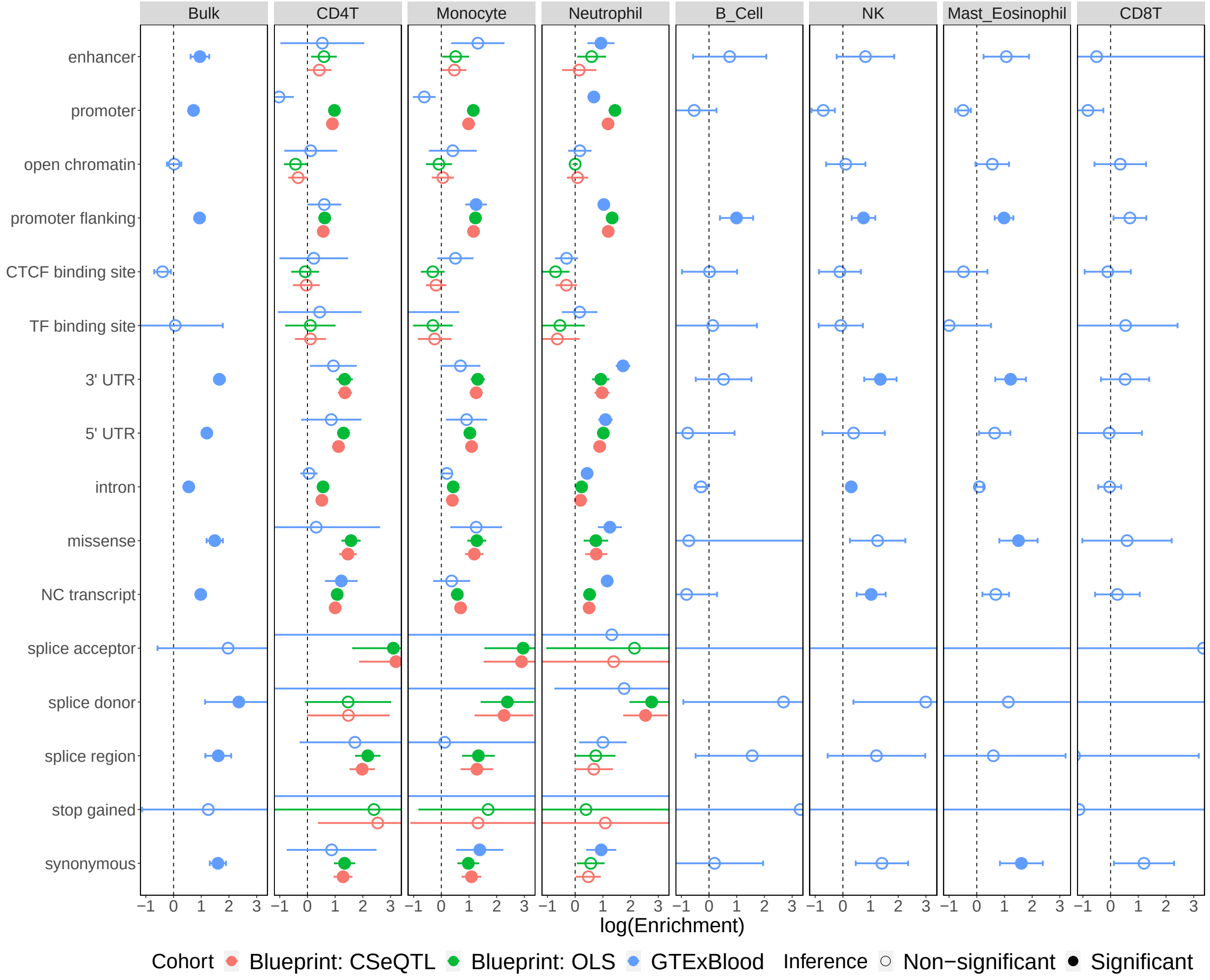

Supplementary Figure 27: **Functional enrichment for the eQTL results from from BLUEPRINT study and GTEx whole blood samples.** Data are presented by point estimate of enrichment (each circle) and the 95% confidence interval (lines centered at point estimates). Nominal p-values are Bonferroni corrected to account for multiple comparison. Filled circles correspond to the ones with lower bound of confidence intervals larger than zero and adjusted p-values < 0.05. Note that for purified RNA-seq data that only contains one cell type, CSeQTL becomes equivalent to TReCASE. The enrichment estimates and their 95% confidence intervals were obtained by Torus with $n = 15,334$ genes.

Comparison	MODEL	CELL TYPE	eGene1; eGene2; eGene intersection	P-value	Correlation All genes	eGenes	non-eGenes
CMC-Ctrl vs. CMC-SCZ	CSeQTL	Bulk	7676; 6828; 5458	0.00e+00	0.71 (0.70, 0.72)	0.76 (0.75, 0.77)	0.26 (0.24, 0.28)
		Astro	644; 502; 177	1.90e-147	0.38 (0.37, 0.40)	0.30 (0.15, 0.43)	0.31 (0.29, 0.33)
		Exc	2246; 1640; 715	7.40e-213	0.37 (0.36, 0.39)	0.31 (0.24, 0.39)	0.26 (0.24, 0.28)
		Inh	1084; 889; 437	7.01e-315	0.35 (0.33, 0.36)	0.28 (0.18, 0.36)	0.23 (0.21, 0.25)
		Micro	197; 186; 36	3.92e-35	0.27 (0.24, 0.29)	0.39 (0.03, 0.66)	0.25 (0.22, 0.27)
		Oligo	381; 256; 66	6.96e-55	0.24 (0.22, 0.26)	0.25 (-0.05, 0.50)	0.20 (0.18, 0.23)
		OPC	375; 265; 68	4.34e-56	0.28 (0.26, 0.30)	0.35 (0.14, 0.53)	0.23 (0.21, 0.25)
	OLS	Bulk	3707; 2872; 2370	0.00e+00	0.61 (0.59, 0.62)	0.76 (0.73, 0.78)	0.26 (0.25, 0.28)
		Astro	0; 0; 0		0.11 (0.09, 0.13)		0.11 (0.09, 0.13)
		Exc	1; 0; 0		0.13 (0.12, 0.15)		0.13 (0.12, 0.14)
		Inh	2; 0; 0		0.12 (0.11, 0.14)		0.12 (0.11, 0.14)
		Micro	0; 0; 0		0.16 (0.14, 0.17)		0.16 (0.14, 0.17)
		Oligo	1; 2; 1	1.32e-04	0.13 (0.11, 0.14)		0.13 (0.11, 0.14)
		OPC	0; 0; 0		0.12 (0.10, 0.13)		0.12 (0.10, 0.13)
CMC-Ctrl vs. GTEx Brain	CSeQTL	Bulk	7194; 7544; 5089	0.00e+00	0.54 (0.53, 0.55)	0.55 (0.53, 0.58)	0.23 (0.20, 0.25)
		Astro	613; 315; 82	1.43e-45	0.23 (0.21, 0.25)	0.34 (0.11, 0.53)	0.19 (0.17, 0.21)
		Exc	2086; 1223; 385	4.33e-45	0.19 (0.17, 0.21)	0.22 (0.12, 0.32)	0.15 (0.13, 0.17)
		Inh	912; 171; 51	2.11e-14	0.15 (0.13, 0.18)	0.11 (-0.18, 0.38)	0.13 (0.11, 0.15)
		Micro	124; 52; 4	5.16e-02	0.13 (0.10, 0.16)	-0.20 (-1.00, 1.00)	0.13 (0.10, 0.16)
		Oligo	373; 78; 15	2.02e-06	0.13 (0.11, 0.15)	0.00 (-0.56, 0.59)	0.13 (0.11, 0.15)
		OPC	310; 106; 9	9.61e-02	0.20 (0.17, 0.22)	0.05 (-0.62, 0.93)	0.18 (0.16, 0.21)
	OLS	Bulk	3447; 2260; 1668	0.00e+00	0.46 (0.45, 0.48)	0.55 (0.51, 0.58)	0.19 (0.17, 0.22)
		Astro	0; 0; 0		0.12 (0.10, 0.14)		0.12 (0.10, 0.14)
		Exc	0; 2; 0		0.12 (0.10, 0.13)		0.12 (0.10, 0.14)
		Inh	2; 0; 0		0.12 (0.10, 0.13)		0.12 (0.10, 0.13)
		Micro	0; 0; 0		0.11 (0.09, 0.13)		0.11 (0.09, 0.13)
		Oligo	0; 0; 0		0.13 (0.12, 0.15)		0.13 (0.12, 0.15)
		OPC	0; 0; 0		0.12 (0.10, 0.14)		0.12 (0.10, 0.14)
CMC-SCZ vs. GTEx Brain	CSeQTL	Bulk	6324; 7472; 4657	0.00e+00	0.54 (0.52, 0.55)	0.54 (0.52, 0.57)	0.23 (0.21, 0.26)
		Astro	460; 310; 66	9.32e-40	0.21 (0.19, 0.23)	0.23 (-0.02, 0.46)	0.18 (0.16, 0.20)
		Exc	1519; 1212; 293	1.22e-38	0.18 (0.17, 0.20)	0.23 (0.12, 0.33)	0.16 (0.14, 0.17)
		Inh	754; 169; 39	3.46e-10	0.14 (0.12, 0.17)	0.29 (-0.09, 0.60)	0.12 (0.10, 0.14)
		Micro	136; 55; 6	3.59e-03	0.12 (0.09, 0.15)	0.31 (-1.00, 1.00)	0.11 (0.08, 0.14)
		Oligo	247; 77; 10	1.33e-04	0.11 (0.09, 0.13)	0.02 (-0.68, 0.60)	0.11 (0.09, 0.13)
		OPC	245; 113; 10	6.06e-03	0.18 (0.15, 0.20)	-0.04 (-0.72, 0.81)	0.16 (0.14, 0.19)
	OLS	Bulk	2596; 2227; 1469	0.00e+00	0.45 (0.44, 0.47)	0.55 (0.50, 0.59)	0.20 (0.18, 0.22)
		Astro	0; 0; 0		0.12 (0.10, 0.13)		0.12 (0.10, 0.13)
		Exc	0; 2; 0		0.13 (0.11, 0.15)		0.13 (0.11, 0.14)
		Inh	0; 0; 0		0.11 (0.09, 0.12)		0.11 (0.09, 0.12)
		Micro	0; 0; 0		0.12 (0.10, 0.13)		0.12 (0.10, 0.13)
		Oligo	1; 0; 0		0.11 (0.09, 0.12)		0.11 (0.09, 0.12)
		OPC	0; 0; 0		0.11 (0.10, 0.13)		0.11 (0.10, 0.13)
BLUEPRINT vs. GTEx Blood	CSeQTL	CD4T	6520; 207; 164	2.35e-02	0.15 (0.13, 0.17)	0.10 (-0.06, 0.24)	0.19 (0.15, 0.23)
		Monocyte	3782; 409; 275	2.71e-09	0.06 (0.04, 0.08)	0.06 (-0.06, 0.17)	0.10 (0.06, 0.13)
		Neutrophil	5322; 3336; 2636	1.99e-160	0.44 (0.42, 0.46)	0.39 (0.36, 0.43)	0.20 (0.16, 0.23)
	OLS	CD4T	3934; 0; 0		0.11 (0.09, 0.13)		0.14 (0.12, 0.17)
		Monocyte	4229; 1; 1	4.02e-01	0.08 (0.06, 0.10)		0.11 (0.09, 0.14)
		Neutrophil	3154; 752; 628	3.54e-170	0.38 (0.36, 0.40)	0.25 (0.17, 0.33)	0.16 (0.13, 0.18)

Supplementary Table 6: **Consistency results between cohorts by model, analysis, and cell type.** Column 4 contains the number of eGenes detected per cohort and shared between cohorts. The fifth column corresponds to the Pearson χ^2 test associating eGene classification between a pair of cohorts. The last three columns contain the Spearman correlation with 95% confidence intervals of minimum p-value per gene across all genes, all shared eGenes, and all shared non-eGenes, respectively.

6.8 Summary of number of eQTL findings with alternative q-value cutoff

DISEASE	MODEL	CELLTYPE	Perm. PVAL	eGenes (%all, %CIS)	overlap_Bulk	ERROR
Control	CSeQTL	Bulk	3.2e-01	13008 (84.8%, 75%)		0.067
		Astro	4.4e-03	1008 (6.6%, 95%)	93%	0.043
		Exc	2.5e-02	3843 (25.3%, 90%)	92%	0.051
		Inh	7.8e-03	1722 (11.5%, 93%)	90%	0.046
		Micro	2.4e-03	411 (2.7%, 90%)	93%	0.050
		Oligo	4.7e-03	764 (5%, 91%)	92%	0.051
		OPC	3.1e-03	606 (4.1%, 95%)	89%	0.043
	OLS	Bulk	4.9e-02	5916 (38.6%, -)		0.050
		Astro	1.7e-05	0 (-, -)	-	0.052
		Exc	7.1e-08	1 (0%, -)	100%	0.053
		Inh	4.1e-07	2 (0%, -)	100%	0.052
		Micro	4.7e-05	0 (-, -)	-	0.056
		Oligo	2.1e-05	4 (0%, -)	50%	0.052
		OPC	2.7e-05	0 (-, -)	-	0.056
SCZ	CSeQTL	Bulk	2.7e-01	12285 (80.4%, 75%)		0.070
		Astro	3.7e-03	809 (5.4%, 93%)	89%	0.046
		Exc	2.4e-02	3381 (22.3%, 89%)	89%	0.055
		Inh	6.2e-03	1411 (9.5%, 94%)	87%	0.049
		Micro	2.2e-03	356 (2.4%, 91%)	84%	0.054
		Oligo	3.2e-03	506 (3.3%, 91%)	89%	0.054
		OPC	2.4e-03	505 (3.4%, 94%)	87%	0.043
	OLS	Bulk	3.4e-02	4675 (30.6%, -)		0.050
		Astro	1.1e-04	0 (-, -)	-	0.058
		Exc	3.0e-05	4 (0%, -)	100%	0.058
		Inh	8.8e-06	0 (-, -)	-	0.056
		Micro	1.7e-05	0 (-, -)	-	0.061
		Oligo	1.5e-05	6 (0%, -)	50%	0.053
		OPC	2.2e-05	0 (-, -)	-	0.056

Supplementary Table 7: **CMC eQTL results given q-value cutoff 0.05.** "Perm. PVAL" is the permutation p-value cutoff for q-value 0.05. eGenes (%all, %CIS) is the number of eGenes, the corresponding percentage of all the genes being eGenes, and the percentage of genes whose most significant eQTL is a cis-eQTLs (defined as those with cis-trans test p-value > 0.01). overlap_Bulk is the percent of eGenes that are also eGenes from bulk eQTL analysis. ERROR is the proportion of eQTLs with p-value smaller than 0.05 using permuted data. All the results are after trimming.

MODEL	CELLTYPE	Perm. PVAL	eGenes (%all, %CIS)	overlap_Bulk	ERROR
CSeQTL	Bulk	5.3e-01	14812 (96.2%, 72%)		0.082
	Astro	6.2e-03	1006 (6.6%, 85%)	99%	0.063
	Exc	3.4e-02	3590 (23.5%, 82%)	97%	0.068
	Inh	2.6e-03	440 (3%, 83%)	98%	0.058
	Micro	1.1e-03	193 (1.3%, 69%)	96%	0.056
	Oligo	1.7e-03	353 (2.4%, 72%)	98%	0.065
	OPC	1.2e-03	257 (1.8%, 81%)	96%	0.050
OLS	Bulk	2.8e-02	4159 (27%, -)		0.051
	Astro	1.1e-04	0 (-, -)	-	0.043
	Exc	1.5e-05	7 (0%, -)	100%	0.050
	Inh	4.6e-05	0 (-, -)	-	0.049
	Micro	1.3e-05	0 (-, -)	-	0.042
	Oligo	1.2e-05	0 (-, -)	-	0.046
	OPC	6.8e-05	0 (-, -)	-	0.045

Supplementary Table 8: **GTEx Brain Frontal Cortex (BA9) eQTL results for q-value cutoff 0.05.** The meaning of the columns are the same as the columns of Supplementary Table 7.

MODEL	CELLTYPE	Perm. PVAL	eGenes (%all, %CIS)	ERROR
CSeQTL		7.2e-01	13256 (99.4%, 70%)	0.084
		1.7e-01	10192 (74.8%, 67%)	0.074
		3.0e-01	9310 (86.5%, 64%)	0.084
OLS		8.6e-02	7256 (54.4%, -)	0.050
		9.0e-02	7611 (55.9%, -)	0.051
		7.8e-02	5608 (52.1%, -)	0.051

Supplementary Table 9: **BLUEPRINT eQTL results for q-value cutoff 0.05.** The meaning of the columns are the same as the columns of Supplementary Table 7.

MODEL	CELLTYPE	Perm. PVAL	eGenes (%all, %CIS)	overlap_Bulk	ERROR
CSeQTL	Bulk	5.2e-01	11517 (95.8%, 80%, -)		0.063
	CD4T	1.7e-03	331 (2.9%, 95%, 0%)	98%	0.040
	CD8T	1.9e-03	317 (2.7%, 97%, 0%)	98%	0.044
	B_Cell	1.4e-03	203 (1.8%, 92%, 0.1%)	98%	0.045
	Monocyte	5.6e-03	678 (5.8%, 86%, 0.4%)	99%	0.056
	NK	4.4e-03	710 (6.1%, 88%, 0.2%)	99%	0.053
	Neutrophil	9.7e-02	6545 (54.6%, 85%, 0.6%)	98%	0.066
	Mast_Eosinophil	1.4e-02	1546 (13.1%, 75%, 1.2%)	97%	0.073
OLS	Bulk	1.1e-01	7426 (61.9%, -, -)		0.050
	CD4T	3.0e-05	0 (-, -, -)	-	0.044
	CD8T	4.2e-05	0 (-, -, -)	-	0.049
	B_Cell	1.6e-05	0 (-, -, -)	-	0.052
	Monocyte	1.3e-05	1 (0%, -, -)	100%	0.059
	NK	3.0e-05	0 (-, -, -)	-	0.055
	Neutrophil	1.6e-02	1882 (16%, -, -)	92%	0.060
	Mast_Eosinophil	1.9e-04	28 (0.2%, -, 0%)	75%	0.067

Supplementary Table 10: **GTEx Whole Blood eQTL results for q-value cutoff 0.05.** The meaning of the columns are the same as the columns of Supplementary Table 7.

6.9 Comparison of eQTL effect directions

Cell type	CSeQTL q-value cutoff	number of eQTLs	Consistency prop. expected	Consistency prop. observed	Chi-squared test pval
All cell types	none	72,790	0.50	0.51	
CD4T	0.1	156	0.51	0.88	1.9e-20
CD4T	0.005	98	0.52	0.92	8.3e-16
CD8T	0.1	137	0.57	0.9	2.9e-18
CD8T	0.005	71	0.62	0.92	4.1e-10
B	0.1	51	0.55	0.88	4.9e-07
B	0.005	31	0.53	0.9	3.6e-05
NK	0.1	212	0.5	0.94	1.4e-36
NK	0.005	145	0.51	0.96	1.9e-27
Monocyte	0.1	159	0.5	0.89	1.3e-22
Monocyte	0.005	121	0.5	0.91	1.3e-18
CD4NC	0.1	90	0.51	0.87	1.3e-11
CD4NC	0.005	54	0.51	0.91	1.4e-08
CD4ET	0.1	66	0.52	0.89	1.2e-09
CD4ET	0.005	44	0.52	0.93	5.5e-08
CD8NC	0.1	43	0.58	0.86	4.9e-05
CD8NC	0.005	21	0.59	0.9	0.0029
CD8ET	0.1	61	0.56	0.89	4.8e-08
CD8ET	0.005	31	0.63	0.9	0.00029
CD8S100B	0.1	33	0.59	0.97	9.1e-07
CD8S100B	0.005	19	0.64	0.95	0.0018
BIN	0.1	26	0.55	0.88	0.00054
BIN	0.005	16	0.56	0.88	0.017
BMem	0.1	25	0.55	0.88	0.0012
BMem	0.005	15	0.51	0.93	0.0043
NKR	0.1	42	0.51	1	7e-10
NKR	0.005	33	0.51	1	7.1e-08
NK	0.1	170	0.51	0.92	1.7e-27
NK	0.005	112	0.53	0.95	4.3e-20
MonoC	0.1	84	0.5	0.89	2e-12
MonoC	0.005	64	0.5	0.91	3.8e-10
MonoNC	0.1	75	0.53	0.89	9.1e-11
MonoNC	0.005	57	0.52	0.91	3.8e-09

Supplementary Table 11: Comparison of eQTL effect directions between CSeQTL results from GTEx whole blood (q-value < 0.1 or 0.005 except the first row where no filtering was conducted) and scRNA-seq eQTL results from Yazar et al. [27] (p-value < 0.01 except for the first row). We considered either the cell types used in CSeQTL or the cell types used by Yazar et al. [27].

Cell type	CSeQTL q-value cutoff	number of eQTLs	Consistency prop. expected	observed	Chi-squared test pval
All cell types	none	39,940	0.50	0.53	
Astro	0.1	149	0.52	0.96	2.9e-28
Astro	0.005	89	0.55	0.97	1.6e-17
Exc	0.1	547	0.54	0.91	2.1e-80
Exc	0.005	284	0.53	0.93	1.6e-46
Inh	0.1	18	0.52	0.78	0.065
Inh	0.005	9	0.52	0.89	0.097
Micro	0.1	13	0.51	0.92	0.012
Micro	0.005	9	0.52	0.89	0.097
Oligo	0.1	62	0.51	0.85	1.3e-07
Oligo	0.005	27	0.52	0.96	1.1e-05
OPC	0.1	15	0.51	0.93	0.0043
OPC	0.005	9	0.59	0.89	0.16

Supplementary Table 12: Comparison of eQTL effect directions between CSeQTL results from GTEx brain and scRNA-seq eQTL results from Bryois et al. [28]. The p-value/q-value cutoffs are the same as Table 11.

Cell type	CSeQTL q-value cutoff	number of eQTLs	Consistency prop. expected	observed	Chi-squared test pval
All cell types	none	35,161	0.50	0.52	
Astro	0.1	53	0.53	0.91	3.5e-08
Astro	0.005	41	0.53	0.93	3.7e-07
Exc	0.1	378	0.54	0.88	6.8e-47
Exc	0.005	229	0.55	0.93	1e-35
Inh	0.1	42	0.53	0.6	0.58
Inh	0.005	27	0.51	0.63	0.32
Micro	0.1	11	0.52	0.82	0.12
Micro	0.005	7	0.53	0.86	0.28
Oligo	0.1	61	0.5	0.89	9.2e-09
Oligo	0.005	28	0.54	0.93	3.9e-05
OPC	0.1	17	0.56	0.65	0.73
OPC	0.005	13	0.63	0.69	1.00

Supplementary Table 13: Comparison of eQTL effect directions between CSeQTL results from CMC schizophrenia patients and scRNA-seq eQTL results from Bryois et al. [28]. The p-value/q-value cutoffs are the same as Table 11.

Cell type	CSeQTL q-value cutoff	number of eQTLs	Consistency prop. expected	observed	Chi-squared test pval
All cell types	none	34,321	0.50	0.52	
Astro	0.1	74	0.5	0.89	7.1e-11
Astro	0.005	55	0.5	0.93	1.2e-09
Exc	0.1	411	0.52	0.83	1.2e-39
Exc	0.005	264	0.51	0.85	4.3e-30
Inh	0.1	58	0.5	0.67	0.017
Inh	0.005	35	0.54	0.69	0.092
Micro	0.1	22	0.51	0.82	0.011
Micro	0.005	15	0.53	0.8	0.094
Oligo	0.1	69	0.57	0.84	4.9e-07
Oligo	0.005	41	0.55	0.83	0.00017
OPC	0.1	15	0.58	0.8	0.15
OPC	0.005	10	0.68	1	0.03

Supplementary Table 14: Comparison of eQTL effect directions between CSeQTL results from CMC controls and scRNA-seq eQTL results from Bryois et al. [28]. The p-value/q-value cutoffs are the same as Table 11.

Supplementary References

[1] Wilson, D.R., Jin, C., Ibrahim, J.G. and Sun, W. ICeD-T provides accurate estimates of immune cell abundance in tumor samples by allowing for aberrant gene expression patterns. *Journal of the American Statistical Association*, 115(531):1055–1065, 2020.

[2] Racle, J., de Jonge, K., Baumgaertner, P., Speiser, D.E. and Gfeller, D. Simultaneous enumeration of cancer and immune cell types from bulk tumor gene expression data. *Elife*, 6:e26476, 2017.

[3] Glastonbury, C.A., Alves, A.C., Moustafa, J.S.E.S. and Small, K.S. Cell-type heterogeneity in adipose tissue is associated with complex traits and reveals disease-relevant cell-specific eQTLs. *The American Journal of Human Genetics*, 104:1013–1024, 2019.

[4] Newman, A.M. et al. Robust enumeration of cell subsets from tissue expression profiles. *Nature methods*, 12(5):453, 2015.

[5] Li, B. et al. Comprehensive analyses of tumor immunity: implications for cancer immunotherapy. *Genome Biology*, 17(1):174, 2016.

[6] Ahn, J. et al. DeMix: deconvolution for mixed cancer transcriptomes using raw measured data. *Bioinformatics*, 29(15):1865–1871, 2013.

[7] Habib, N. et al. Massively parallel single-nucleus RNA-seq with DroNc-seq. *Nature methods*, 14(10):955, 2017.

[8] Hodge, R.D. et al. Conserved cell types with divergent features in human versus mouse cortex. *Nature*, 573(7772):61–68, 2019.

[9] McCarthy, D.J., Campbell, K.R., Lun, A.T.L. and Wills, Q.F. Scater: pre-processing, quality control, normalisation and visualisation of single-cell RNA-seq data in R. *Bioinformatics*, 33:1179–1186, 2017.

[10] Lun, A.T., McCarthy, D.J. and Marioni, J.C. A step-by-step workflow for low-level analysis of single-cell RNA-seq data with bioconductor. *F1000Res.*, 5:2122, 2016.

[11] Smyth, G.K. Limma: linear models for microarray data. In *Bioinformatics and computational biology solutions using R and Bioconductor*, pages 397–420. Springer, 2005.

[12] Krijthe, J., van der Maaten, L. and Krijthe, M.J. Package 'rtsne', 2018.

[13] Finak, G. et al. MAST: a flexible statistical framework for assessing transcriptional changes and characterizing heterogeneity in single-cell RNA sequencing data. *Genome biology*, 16(1):278, 2015.

[14] Sun, W. A statistical framework for eQTL mapping using RNA-seq data. *Biometrics*, 68(1):1–11, 2012.

[15] Hu, Y.J., Sun, W., Tzeng, J.Y. and Perou, C.M. Proper use of allele-specific expression improves statistical power for cis-eQTL mapping with RNA-seq data. *Journal of the American Statistical Association*, 110(511):962–974, 2015.

[16] Zhabotynsky, V. et al. eQTL mapping using allele-specific count data is computationally feasible, powerful, and provides individual-specific estimates of genetic effects. *PLoS Genetics*, 18(3):e1010076, 2022.

[17] Storey, J.D. and Tibshirani, R. Statistical significance for genomewide studies. *Proceedings of the National Academy of Sciences*, 100(16):9440–9445, 2003.

[18] Fromer, M. et al. Gene expression elucidates functional impact of polygenic risk for schizophrenia. *Nature neuroscience*, 19(11):1442, 2016.

[19] Hoffman, G.E. et al. CommonMind Consortium provides transcriptomic and epigenomic data for Schizophrenia and Bipolar Disorder. *Scientific data*, 6(1):1–14, 2019.

[20] Chen, L. et al. Genetic drivers of epigenetic and transcriptional variation in human immune cells. *Cell*, 167(5):1398–1414, 2016.

[21] Delaneau, O., Coulonges, C. and Zagury, J.F. Shape-IT: new rapid and accurate algorithm for haplotype inference. *BMC bioinformatics*, 9(1):540, 2008.

[22] Howie, B.N., Donnelly, P. and Marchini, J. A flexible and accurate genotype imputation method for the next generation of genome-wide association studies. *PLoS genetics*, 5(6):e1000529, 2009.

[23] Picard toolkit, 2019.

[24] Sun, W., Zhabotynsky, V. and Jin, C. *asSeq: allele specific sequencing study*, 2018. R package version 0.99.501.

[25] GTEx Consortium. The GTEx Consortium atlas of genetic regulatory effects across human tissues. *Science*, 369(6509):1318–1330, 2020.

[26] Leek, J.T. and Storey, J.D. Capturing heterogeneity in gene expression studies by surrogate variable analysis. *PLoS genetics*, 3(9), 2007.

[27] Yazar, S. et al. Single-cell eqtl mapping identifies cell type–specific genetic control of autoimmune disease. *Science*, 376(6589):eabf3041, 2022.

[28] Bryois, J. et al. Cell-type-specific cis-eqtls in eight human brain cell types identify novel risk genes for psychiatric and neurological disorders. *Nature Neuroscience*, 25(8):1104–1112, 2022.

[29] Hafemeister, C. and Satija, R. Normalization and variance stabilization of single-cell rna-seq data using regularized negative binomial regression. *Genome biology*, 20(1):1–15, 2019.
